# Total Synthesis of the Four Stereoisomers of Cyclo(l-Trp-l-Arg) Raises Uncertainty of the Structures of the Natural Products and Invalidates Their Promising Antimicrobial Activities

**DOI:** 10.3390/molecules27185913

**Published:** 2022-09-12

**Authors:** Dan Chen, Daniel J. Park, Melissa M. Cadelis, Hana Douafer, Marie Lise Bourguet-Kondracki, Jean Michel Brunel, Brent R. Copp

**Affiliations:** 1 School of Chemical Sciences, The University of Auckland, Waipapa Taumata Rau, Private Bag 92019, Auckland 1142, New Zealand; 2 UMR MD1 “Membranes et Cibles Thérapeutiques”, U1261 INSERM, Faculté de Pharmacie, Aix-Marseille Universite, 27 bd Jean Moulin, 13385 Marseille, France; 3 Laboratoire Molécules de Communication et Adaptation des Micro-organismes, UMR 7245 CNRS, Muséum National d’Histoire Naturelle, 57 rue Cuvier (C.P. 54), 75005 Paris, France

**Keywords:** diketopiperazine, cyclo(Trp-Arg), natural product, synthesis, structure revision, antimicrobial, antibiotic enhancement

## Abstract

New therapeutic options to combat the growing incidence of antimicrobial resistance are urgently needed. A 2015 publication reported the isolation and biological evaluation of two diketopiperazine natural products, cyclo(l-Trp-l-Arg) (CDP 2) and cyclo(d-Trp-d-Arg) (CDP 3), from an *Achromobacter* sp. bacterium, finding that the latter metabolite in particular exhibited strong antibacterial activity towards a range of wound-related microorganisms and could synergize the action of ampicillin. Intrigued by these biological activities and noting inconsistencies in the structural characterization of the natural products, we synthesized the four diastereomers of cyclo(Trp-Arg) and evaluated them for antimicrobial and antibiotic enhancement properties. The detailed comparison of spectroscopic data raises uncertainty regarding the structure of CDP 2 and disproves the structure of CDP 3. In our hands, none of the four stereoisomers of cyclo(Trp-Arg) exhibited detectable intrinsic antimicrobial properties towards a range of Gram-positive and Gram-negative bacteria or fungi nor could they potentiate the action of antibiotics. These discrepancies in biological properties, compared with the activities reported in the literature, reveal that these specific cyclic dipeptides do not represent viable templates for the development of new treatments for microbial infections.

## 1. Introduction

The identification of new antimicrobials is becoming more urgent with the growing incidence and prevalence of antimicrobial resistance [1]. While natural products represented some of the most central examples of antibiotics in the so-called “Golden Age” of antibiotics, interest in their discovery and development by major pharmaceutical companies fell to the wayside in the 1980s [2]. The overall lack of success that followed the subsequent focus on combinatory chemistry driven by target-specific screening has led to a revival of phenotypic-based screening that encompasses investigation of libraries that include natural products [3,4,5].

**Figure 1:**
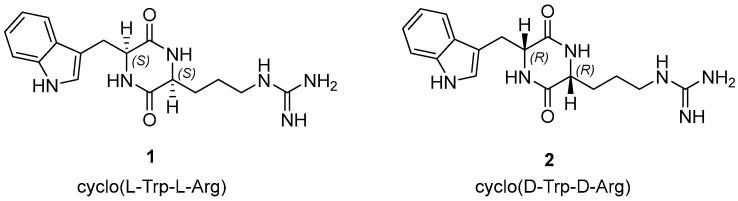
Proposed structures of CDP 2 (**1**) and CDP 3 (**2**) [8].

In continuation of our ongoing interest in the discovery and development of new antimicrobials and antibiotic enhancers [6,7], we were interested in a publication reporting the structures of three diketopiperazines from the bacterium *Achromobacter* sp. [8], two of which were claimed to be the enantiomers cyclo(l-trp-l-Arg) (CDP 2, **1**) and cyclo(d-trp-d-Arg) (CDP 3, **2**) (Figure 1). While the former natural product was only mildly active, the latter exhibited pronounced growth inhibition of a range of Gram-positive and Gram-negative bacteria with MIC 0.5–64 μg/mL. Especially of interest to us was the observation of synergism for either compound in combination with ampicillin towards a range of wound associated bacteria. Of note was the exceptionally low checkerboard assay FICI value (0.09) for the ampicillin + **2** combination towards the Gram-negative bacterium *Pseudomonas aeruginosa*. Overall, these results suggested that compounds **1** and **2** could represent a template from which to develop new examples of antimicrobial and antibiotic enhancing compounds.

Before commencing an expansive structure–activity relationship study, a closer inspection of the structure elucidation and characterization of natural products **1** and **2** raised some inconsistencies:

1. While **1** and **2** were reported to be enantiomers, supported by the observation of similar magnitude and opposite sign specific rotations of +145 and −167, and essentially identical melting points ranges of 265.1–267.34 °C and 262.23–265.58 °C, respectively, they were reported to have different ^1^H and ^13^C NMR chemical shifts in the same (achiral) solvent (DMSO-*d*_6_).
2. Although analyzed for purity using an achiral C18 column HPLC system, compounds **1** and **2** exhibited different retention times of 20.241 min and 11.232 min, respectively.
3. Stereochemistry was attributed using standard Marfey’s analysis, but no HPLC traces were presented—the results were ‘data not shown’.
4. Molecular formulae (C_17_H_23_N_6_O_2_) for **1** and **2** were assigned by analysis of HRESIMS [M + H]^+^ data, but the reported observed values of *m*/*z* 343.39558 and 343.37431 were significantly different from the expected exact mass of *m*/*z* 343.18770 (Bruker Compass DataAnalysis v 4.1) (with mass errors of 606 ppm and 544 ppm, respectively being far in excess of the acceptable 4–5 ppm error range) and are in fact closer to the average mass value of 343.41405.
5. The experimental section states that the natural products were purified using silica gel column chromatography eluting with dichloromethane-hexane and ethyl acetate-dichloromethane solvent mixtures, followed by crystallization using hexane and benzene. These conditions are particularly suited to the purification of non-polar natural products; however, given the basic nature of the guanidine group (pKa~12.5), these natural products would have been isolated as salts (of undefined counterion) and would not have eluted from a silica column under the stated conditions.

These inconsistencies raised questions as to the proposed structures of the bacterial-derived diketopiperazine natural products cyclo(l-Trp-l-Arg) **1** and cyclo(d-Trp-d-Arg) **2**. Therefore, we undertook the total synthesis and characterization of the four stereoisomers of cyclo(Trp-Arg) and evaluation of their antimicrobial and antibiotic-enhancing properties. Herein, we report the results of these studies that have led us to question the structure of CDP2 and disprove the structure of CDP3. A lack of detectable biological activity of the four synthetic stereoisomers reveals that these molecules have no potential to act as templates for new antibiotics or adjuvants.

## 2. Results and Discussion

The synthesis of all four stereoisomers of cyclo(Trp-Arg) has been previously reported, without experimental details [9,10], and comprehensively characterized using NMR and combinations of vibrational and electronic circular dichroism. Unfortunately, the NMR solvents and chiroptical techniques used by those authors were different to those used to characterize natural products **1** and **2** preventing direct comparison.

**Scheme 1:**
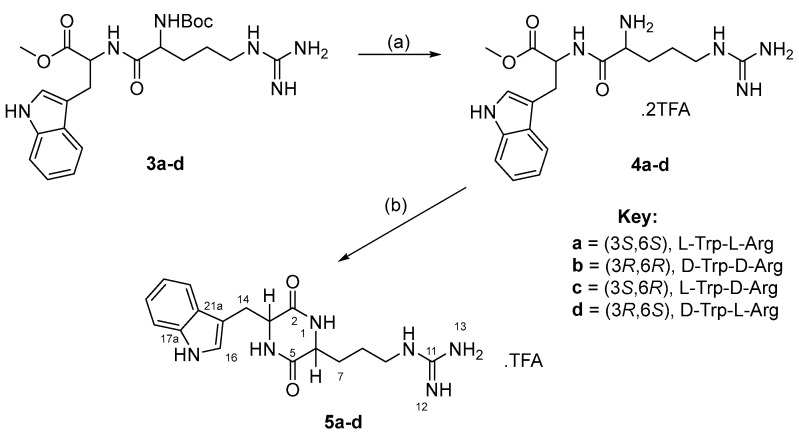
General synthetic route for the synthesis of cyclo(Trp-Arg) stereoisomers. Reagents and conditions*:* (**a**) TFA (0.2 mL) in CH_2_Cl_2_ (2 mL), N_2_, 2 h (55–69%); (**b**) NH_4_OH in MeOH, rt, 24 h (47–71%).

We chose to synthesize the four stereoisomers of cyclo(Trp-Arg) using the general procedure shown in Scheme 1. Coupling of Trp-OMe with *N*α-Boc-Arg mediated by (2-(1*H*-benzotriazol-1-yl)-1,1,3,3-tetramethyluronium hexafluorophosphate (HBTU)/hydroxybenzotriazole (HOBt) afforded protected dipeptides **3a–d** (yields of 56–91%), which were then subjected to reaction with trifluoroacetic acid in dichloromethane to give dipeptide methylesters **4a–d** as the di-TFA salt. Incubation of **4a–d** with NH_4_OH in MeOH [11] at room temperature for 24 h provided, after purification using C8 reversed-phase column chromatography, the target cyclo(Trp-Arg) diketopiperazines **5a–d** as the presumed mono TFA salt. Little to no evidence was observed for the formation of racemization products during the mild conditions used for the diketopiperazine ring closure.

**Figure 2:**
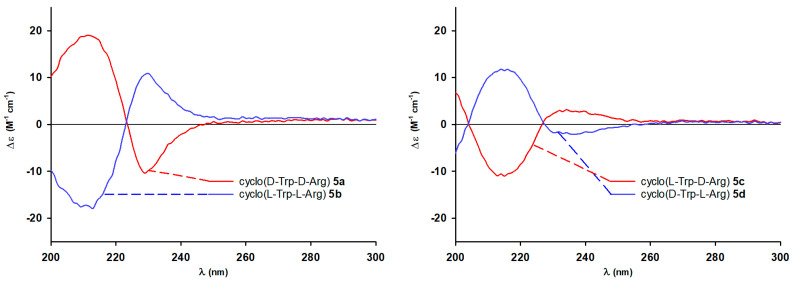
Electronic circular dichroism spectra observed for diketopiperazines **5a** and **5b** (**left**) and **5c** and **5d** (**right**).

Each of the diastereomers **5a–d** was characterized by ESI mass spectrometry, chiroptically and by NMR, with complete 1-D and 2-D data sets acquired separately in DMSO-*d*_6_ and CD_3_OD (see Supporting Information Figures S1–S8 for ^1^H and ^13^C spectra). As anticipated, all four stereoisomers exhibited essentially the same ESIMS protonated molecular ion ([M + H]^+^ *m*/*z* 343.1870 ± 0.0003 (0.9–2.9 ppm error), which exact mass matched to the anticipated molecular formula (see Experimental). Chiroptical characterization of the four diastereomers used a combination of specific rotation and electronic circular dichroism (ECD). Enantiomer pairs **5a**,**b** and **5c**,**d** exhibited equal magnitude and opposite sign using both techniques, while diastereomeric pairs (**5a**,**c** and **5b**,**d**) were noticeably different (specific rotation values given in Table 1, ECD spectra shown in Figure 2).

Our results were consistent with two previous studies of cyclo(Trp-Arg), with [α]_D_ for **5a** being close to the value reported by Sasaki et al. for cyclo(l-Trp-l-Arg) (lit. [12] −13 (H_2_O) vs. **5a** [α]_D_ (MeOH) −10.5), and with the ECD Cotton effects and Dε values in close agreement with those reported for cyclo(l-Trp-l-Arg) and cyclo(l-Trp-d-Arg) by Li et al. [9].

Enantiomeric pairs of the diketopiperazines also exhibited identical ^1^H and ^13^C NMR spectra while subtle differences were observed between diastereomers. As described at length by Li et al., *syn* diastereomers of cyclo(Trp-Arg) **5a** and **5b**, i.e., those with the tryptophan and arginine sidechains on the same face of the diketopiperazine ring, exhibit diagnostically shielded chemical shifts for arginine sidechain protons H_2_-7 and H_2_-8 (δ_H_ 0.87–0.49) versus the corresponding *anti* diastereomers **5c** and **5d** (H_2_-7 and H_2_-8, δ_H_ 1.67–1.44). Careful comparison of our data with those previously reported by Li et al. [9] showed good to excellent agreement (as measured by mean absolute error (MAE) values [13]) with ^1^H and ^13^C chemical shifts (CD_3_OD, plus ^1^H exchangeables observed in DMSO-*d*_6_) for **5a**,**b** (^1^H MAE 0.014; ^13^C MAE 0.02) and **5c**,**d** (^1^H MAE 0.005; ^13^C MAE 0.00) (Tables S1 and S2).

Safe in the knowledge that our spectroscopic data observed for **5a–d** were in agreement with previously reported data for the same compounds, we then compared our data with those reported by Deepa et al. for **1** and **2**. This analysis is summarized as follows:

1. ^1^H NMR. The ^1^H chemical shifts reported for H_2_-7 and H_2_-8 of the natural products **1** and **2** were in the shielded region with δ_H_ 0.81–0.54 (**1**) and δ_H_ 0.88–0.61 (**2**), consistent with both having *syn* substitution on the diketopiperazine ring as claimed. While stated to have been recorded in DMSO-*d*_6_ solvent, neither set of ^1^H NMR data reported for **1** and **2** agreed with our data acquired for LL/DD (**5a**,**b**) in DMSO-*d*_6_ (**1** vs. **5a**,**b** MAE 0.12, **2** vs. **5a**,**b** MAE 0.17) (Tables S3 and S4). A better match (based upon lowest MAE) was found for **1** when compared to data acquired in CD_3_OD solvent with the addition of ^1^H exchangeable shifts reported from DMSO-*d*_6_ data (**1** vs. **5a**,**b** MAE 0.05, **2** vs. **5a**,**b** MAE 0.11) (Tables S3 and S4). Thus, we concluded that **1** was indeed a diketopiperazine bearing *syn* disubstitution but that the NMR data had actually been acquired in CD_3_OD with exchangeable ^1^H chemical shifts determined in DMSO-*d*_6_ solvent.
2. ^13^C NMR. Similar comparison of ^13^C NMR data reported for **1** and **2** with the chemical shifts acquired for **5a**,**b** in either CD_3_OD or DMSO-*d*_6_ identified the closest fit to be for natural product CDP 2 **1** and our CD_3_OD solvent data set (MAE 0.1) (Tables S5 and S6).

Based upon our analysis of ^1^H and ^13^C NMR data, we could conclude that natural product **1** was indeed a *syn*-substituted diketopiperazine (as claimed) [8] but that the original NMR data was acquired in CD_3_OD and using DMSO-*d*_6_ solvent to observe the exchangeable NH proton signals. Given that enantiomers exhibit identical NMR data (when acquired in achiral solvents), we are not able to assign a structure or relative configuration to natural product CDP3 **2** at this time.

3. Specific rotation. With the preceding analysis of NMR data suggesting natural product **1** was indeed a *syn*-substituted diketopiperazine, we next used comparison of specific rotation values to assign absolute configuration. A large magnitude dextrorotatory value was reported for **1** ([α]_D_ (*c* 0.02, MeOH) +145 while we observed low magnitude values of −10.5 (for **5a**) and +10.7 (for **5b**). A previously reported specific rotation for cyclo(l-Trp-l-Arg) (hydrochloride salt) of −13 (H_2_O) [12] is in good agreement with our data. These results revealed a disconnect between the specific rotation and peptide hydrolysis results reported for CDP 2 [8] and our data but, as we did not have access to authentic samples of the natural products, we were unable to determine which information reported for the natural product is incorrect. We conclude that there remains uncertainty regarding the absolute configuration of CDP 2.

In their natural product isolation paper, Deepa et al. reported that diketopiperazines **1** and **2** exhibited modest to moderate levels of activity towards a panel of wound-associated bacterial pathogens including *Staphylococcus aureus* (**1**, MIC 64 μg/mL; **2**, 0.5 μg/mL), *P. aeruginosa* (**1**, MIC 250 μg/mL; **2**, 0.5 μg/mL) and *Klebsiella pneumonia* (**1**, MIC 125 μg/mL; **2**, 2 μg/mL) [8]. The intrinsic antimicrobial activity of **5a–d** was evaluated against a range of Gram-positive (*S. aureus* and MRSA) and Gram-negative (*Escherichia coli*, *P. aeruginosa*, *K. pneumoniae* and *Acinetobacter baumannii*) bacteria and two fungal strains (*Candida albicans* and *Cryptococcus neoformans*). Cytotoxicity towards HEK293 (human kidney epithelial cell line) and hemolytic activity against human red blood cells were also determined. In all cases, our synthetic samples **5a–d** were found to be biologically inactive, with no detectable growth inhibition at the highest test concentrations of 350 μg/mL (for *S. aureus*, *E. coli* and *P. aeruginosa*) and 32 μg/mL (for all other assays) (data not shown).

While the structure assignment was confirmed, the synthetically-derived materials did not possess any antibacterial activity nor any doxycycline or ampicillin-enhancing properties towards *P. aeruginosa* PAO1 when tested at a range of concentrations up to 400 μM as previously reported for the natural products [8]. Unfortunately, the originally isolated natural product material was not available for direct comparison with the synthetic compounds. Our results revealed that these diketopiperazine derivatives are not antibacterial agents or antibiotic enhancers.

## 3. Materials and Methods

### 3.1. Chemical Synthesis General Methods

Mass spectra were recorded using a MicrOTOF-QII mass spectrometer (Bruker Daltonics, Bremen, Germany) coupled with a KD Scientific syringe pump, with analysis using Bruker Compass DataAnalysis v 4.1 software. Infrared spectra were recorded on a Perkin Elmer Spectrum 100 Fourier Transform infrared spectrometer equipped with a universal ATR accessory. Optical rotations were obtained with a Rudolph Analytical Autopol IV automatic polarimeter using a 0.1 dm cell (concentration units of g/100 mL). Electronic circular dichroism readings were obtained with a Chirascan circular dichroism spectrometer using a 1 mm cuvette (concentration units of mol/L). All NMR spectra were recorded using a Bruker (Karlsruhe, Germany) Avance 400 spectrometer operating at 400.13 for ^1^H nuclei and 100.62 for ^13^C nuclei. Chemical shifts are expressed in parts per million (ppm) relative to the solvent peaks (DMSO-*d*_6_: ^1^H 2.50, ^13^C 39.52 ppm; CD_3_OD: ^1^H 3.31, ^13^C 49.00 ppm). Assignments are based on 1- and 2-dimensional NMR experiments and analogue comparisons. Standard Bruker pulse sequences were utilized. Reversed-phase flash column chromatography was carried out using LiChroPrep RP-8 (40–63 μm) (Merck Millipore, Darmstadt, Germany). Analytical thin layer chromatography (TLC) was carried out on 0.2 mm thick plates of Merck DC Kieselgel 60 RP-18 F254S plates. All solvents were of analytical grade or better and/or purified according to standard procedures. Chemical reagents used were purchased from standard chemical suppliers and used as purchased.

#### 3.1.1. General Procedure A: Amide Bond Formation

HBTU was added to a stirred solution of *N*α-Boc-Arg-OH hydrochloride (1.05 eq), Trp-OMe (1.0 eq.), HOBt (3.6 eq.), and diisopropylethylamine (DIPEA) (4.8 eq.) in anhydrous DMF (2 mL) at 0 °C (1.2 eq.). The reaction mixture was stirred for 1.5 h under N_2_ atmosphere and then ethyl acetate (50 mL) was added and the organic layer was washed with citric acid (100 mL), sat. NaHCO_3_ (100 mL) and brine (100 mL), then dried with anhydrous MgSO_4_. The organic layer was then dried in vacuo before being taken to the next step without further purification.

#### 3.1.2. General Procedure B: Boc Deprotection

A solution of the *tert*-butyl-carbamate derivative was stirred in CH_2_Cl_2_ (2 mL) with TFA (0.2 mL) at room temperature under N_2_ for 2 h, then dried in vacuo. The crude product was purified using C_8_ reversed-phase column chromatography (MeOH (+0.05% TFA):H_2_O (+0.05% TFA), 0:100 ⟶ 1:3) to afford the product as the di-TFA salt.

#### 3.1.3. General Procedure C: Diketopiperazine Formation

NH_4_OH was added dropwise to a solution of deprotected dipeptide in MeOH (0.25 M) at 0 °C (28–30% in H_2_O, 1 mL per 6 mL MeOH). The reaction mixture was stirred for 24 h after which the crude product was purified using C8 reversed-phase column chromatography eluting with water.

#### 3.1.4. Methyl (*tert*-butoxycarbonyl)-l-arginyl-l-tryptophanate hydrochloride (**3a**)

Following general procedure A, reaction of *N*α-Boc-l-Arg-OH (56 mg, 0.206 mmol), l-tryptophan methyl ester hydrochloride (50 mg, 0.196 mmol), HOBt (96 mg, 0.706 mmol), DIPEA (0.16 mL, 0.941 mmol), and HBTU (89 mg, 0.235 mmol) afforded the hydrochloride salt of dipeptide **3a** as a clear oil/gum (91 mg, 91%). [α]^21^_D_ +0.77 (*c* 0.130, MeOH); R*_f_* = 0.09 (MeOH); IR (ATR) *v*_max_ 3362, 2952, 2844, 1738, 1659, 1524, 1162, 1016, 824 cm^−1^; ^1^H NMR (400 MHz, DMSO-*d*_6_) δ 10.92–10.87 (1H, m, NH-5), 8.20 (1H, d, *J* = 7.2 Hz, NH-11), 7.72–7.62 (1H, m, NH-17), 7.48 (1H, d, *J* = 7.5 Hz, H-9), 7.34 (1H, d, *J* = 7.5 Hz, H-6), 7.18–7.14 (1H, m, H-4), 7.09–7.03 (1H, m, H-7), 7.01–6.95 (1H, m, H-8), 6.88 (1H, d, *J* = 8.1 Hz, NH-21), 4.53 (1H, dt, *J* = 6.8, 6.8 Hz, H-1), 4.02–3.94 (1H, m, H-13), 3.54 (3H, br s, OMe), 3.15–3.10 (1H, m, H-2a), 3.10–3.06 (1H, m, H-2b), 3.08–3.02 (2H, m, H_2_-16), 1.67–1.55 (1H, m, H-14a), 1.53–1.41 (3H, m, H-14b, H_2_-15), 1.37 (9H, br s, Boc); ^13^C NMR (100 MHz, DMSO-*d*_6_) δ 172.1 (C-10/C-12), 172.0 (C-10/C-12), 156.7 (C-18), 155.2 (Boc), 136.1 (C-5a), 127.0 (C-9a), 123.7 (C-4), 121.0 (C-7), 118.4 (C-8), 117.9 (C-9), 111.4 (C-6), 109.1 (C-3), 78.2 (Boc), 53.6 (C-13), 53.0 (C-1), 51.8 (OMe), 40.4 (C-16), 29.1 (C-14), 28.2 (Boc), 27.0 (C-2), 25.0 (C-15); (+)-HRESIMS [M + H]^+^ *m*/*z* 475.2647 (calcd for C_23_H_35_N_6_O_5_, 475.2663).

#### 3.1.5. Methyl (*tert*-butoxycarbonyl)-d-arginyl-d-tryptophanate hydrochloride (**3b**)

Following general procedure A, reaction of *N*α-Boc-d-Arg-OH hydrochloride (64 mg, 0.206 mmol), d-Trp-OMe hydrochloride (50 mg, 0.196 mmol), HOBt (95 mg, 0.706 mmol), DIPEA (0.16 mL, 0.941 mmol), and HBTU (89 mg, 0.235 mmol) afforded the hydrochloride salt of dipeptide **3b** as a clear oil/gum (56 mg, 56%). [α]^21^_D_ −0.74 (*c* 0.136, MeOH); R*_f_* = 0.09 (MeOH); IR (ATR) *v*_max_ 3364, 2953, 2837, 1738, 1658, 1520, 1161, 839 cm^−1^; ^1^H NMR (400 MHz, DMSO-*d*_6_) δ 10.92–10.82 (1H, m, NH-5), 8.20 (1H, d, *J* = 7.0 Hz, NH-11), 7.49 (1H, d, *J* = 8.1 Hz, H-9), 7.35 (1H, d, *J* = 8.1 Hz, H-6), 7.20–7.16 (1H, m, H-4), 7.11–7.05 (1H, m, H-7), 7.03–6.97 (1H, m, H-8), 6.88 (1H, d, *J* = 8.1 Hz, NH-21), 4.59–4.50 (1H, m, H-1), 4.05–3.96 (1H, m, H-13), 3.56 (3H, br s, OMe), 3.16–3.11 (1H, m, H-2a), 3.11–3.04 (3H, m, H-2b, H_2_-16), 1.68–1.57 (1H, m, H-14a), 1.55–1.42 (3H, m, H-14b, H_2_-15), 1.39 (9H, br s, Boc); ^13^C NMR (100 MHz, DMSO-*d*_6_) δ 172.1 (C-10/C-12), 172.0 (C-10/C-12), 156.7 (C-18), 155.2 (Boc), 136.1 (C-5a), 127.0 (C-9a), 123.7 (C-4), 121.0 (C-7), 118.4 (C-8), 117.9 (C-9), 111.4 (C-6), 109.1 (C-3), 78.2 (Boc), 53.6 (C-13), 53.0 (C-1), 51.8 (OMe), 40.4 (C-16), 29.1 (C-14), 28.2 (Boc), 27.0 (C-2), 25.0 (C-15); (+)-HRESIMS [M + H]^+^ *m*/*z* 475.2650 (calcd for C_23_H_35_N_6_O_5_, 475.2663).

#### 3.1.6. Methyl (*tert*-butoxycarbonyl)-d-arginyl-l-tryptophanate hydrochloride (**3c**)

Following general procedure A, reaction of *N*α-Boc-d-Arg-OH hydrochloride (64 mg, 0.206 mmol), l-Trp-OMe hydrochloride (50 mg, 0.196 mmol), HOBt (95 mg, 0.706 mmol), DIPEA (0.16 mL, 0.941 mmol), and HBTU (89 mg, 0.235 mmol) afforded the hydrochloride salt of dipeptide **3c** as a clear oil/gum (67 mg, 67%). [α]^21^_D_ +0.98 (*c* 0.102, MeOH); R*_f_* = 0.09 (MeOH); IR (ATR) *v*_max_ 3357, 2954, 1731, 1653, 1516, 1368, 1161, 837 cm^−1^; ^1^H NMR (400 MHz, DMSO-*d*_6_) δ 10.86 (1H, d, *J* = 2.0 Hz, NH-5), 8.17 (1H, d, *J* = 7.9 Hz, NH-11), 7.48 (1H, d, *J* = 7.9 Hz, H-9), 7.40 (1H, t, *J* = 5.2 Hz, NH-17), 7.34 (1H, d, *J* = 7.9 Hz, H-6), 7.12 (1H, d, *J* = 2.0 Hz, H-4), 7.07 (1H, ddd, *J* = 8.2, 7.9, 1.0 Hz, H-7), 6.99 (1H, ddd, *J* = 8.2, 7.9, 1.0 Hz, H-8), 6.84 (1H, d, *J* = 8.2 Hz, NH-21), 4.51 (1H, dt, *J* = 8.6, 6.2 Hz, H-1), 3.97 (1H, dt, *J* = 9.2, 5.9 Hz, H-13), 3.58 (3H, br s, OMe), 3.20–3.11 (1H, m, H-2a), 3.10–3.04 (1H, m, H-2b), 3.02–2.96 (2H, m, H_2_-16), 1.58–1.47 (1H, m, H-14a), 1.47–1.37 (1H, m, H-14b), 1.38 (9H, br s, Boc), 1.37–1.28 (2H, m, H_2_-15); ^13^C NMR (100 MHz, DMSO-*d*_6_) δ 172.1 (C-10/C-12), 171.8 (C-10/C-12), 156.6 (C-18/Boc), 155.2 (C-18/Boc), 136.1 (C-5a), 127.0 (C-9a), 123.7 (C-4), 121.0 (C-7), 118.4 (C-8), 117.9 (C-9), 111.4 (C-6), 109.2 (C-3), 78.2 (Boc), 53.6 (C-13), 52.9 (C-1), 51.8 (OMe), 40.4 (C-16), 29.1 (C-14), 28.2 (Boc), 27.2 (C-2), 24.9 (C-15); (+)-HRESIMS [M + H]^+^ *m*/*z* 475.2651 (calcd for C_23_H_35_N_6_O_5_, 475.2663).

#### 3.1.7. Methyl (*tert*-butoxycarbonyl)-l-arginyl-d-tryptophanate hydrochloride (**3d**)

Following general procedure A, reaction of *N*α-Boc-l-Arg-OH (113 mg, 0.413 mmol), d-Trp-OMe hydrochloride (100 mg, 0.393 mmol), HOBt (190 mg, 1.41 mmol), DIPEA (0.33 mL, 1.89 mmol), and HBTU (179 mg, 0.472 mmol) afforded the hydrochloride salt of dipeptide **3d** as a clear oil/gum (164 mg, 82%). [α]^19^_D_ +3.0 (*c* 0.10, MeOH); R*_f_* = 0.11 (MeOH); IR (ATR) *v*_max_ 3363, 2952, 2834, 1738, 1654, 1520, 1440, 1392, 1017, 841 cm^−1^; ^1^H NMR (400 MHz, DMSO-*d*_6_) δ 10.88 (1H, d, *J* = 1.9 Hz, NH-5), 8.18 (1H, d, *J* = 7.9 Hz, NH-11), 7.48 (1H, d, *J* = 7.5 Hz, H-9), 7.48–7.42 (1H, m, NH-17), 7.34 (1H, d, *J* = 7.5 Hz, H-6), 7.12 (1H, d, *J* = 1.9 Hz, H-4), 7.07 (1H, ddd, *J* = 8.4, 7.5, 1.2 Hz, H-7), 6.99 (1H, ddd, *J* = 8.4, 7.5, 1.2 Hz, H-8), 6.83 (1H, d, *J* = 8.3 Hz, NH-21), 4.51 (1H, dt, *J* = 7.5, 6.4 Hz, H-1), 3.98 (1H, dt, *J* = 6.2, 5.3 Hz, H-13), 3.58 (3H, br s, OMe), 3.19–3.12 (1H, m, H-2a), 3.10–3.03 (1H, m, H-2b), 3.03–2.97 (2H, m, H_2_-16), 1.58–1.46 (1H, m, H-14a), 1.46–1.36 (1H, m, H-14b), 1.40–1.28 (2H, m, H_2_-15), 1.38 (9H, br s, Boc); ^13^C NMR (100 MHz, DMSO-*d*_6_) δ 172.1 (C-10/C-12), 171.8 (C-10/C-12), 156.6 (C-18), 155.2 (Boc), 136.1 (C-5a), 127.0 (C-9a), 123.7 (C-4), 121.0 (C-7), 118.4 (C-8), 117.9 (C-9), 111.4 (C-6), 109.2 (C-3), 78.2 (Boc), 53.6 (C-13), 52.9 (C-1), 51.8 (OMe), 40.4 (C-16), 28.3 (C-14, Boc), 27.2 (C-2), 24.9 (C-15); (+)-HRESIMS [M + H]^+^ *m*/*z* 475.2652 (calcd for C_23_H_35_N_6_O_5_, 475.2663).

#### 3.1.8. Methyl l-arginyl-l-tryptophanate bis(2,2,2-trifluoroacetate) (**4a**)

Following general procedure B, dipeptide **3a** (49 mg, 0.096 mmol) was reacted with TFA (0.2 mL) in CH_2_Cl_2_ (2 mL) to afford the di-TFA salt of dipeptide **4a** a pale yellow oil/gum (33 mg, 57%). [α]^21^_D_ +1.5 (*c* 0.135, MeOH); R*_f_* = 0.35 (MeOH); IR (ATR) *v*_max_ 3350, 3199, 3067, 2958, 2879, 1667, 1630, 1532, 1200, 1135 cm^−1^; ^1^H NMR (400 MHz, DMSO-*d*_6_) δ 10.95 (1H, d, *J* = 2.4 Hz, NH-5), 8.95 (1H, d, *J* = 7.4 Hz, NH-11), 8.20 (3H, d, *J* = 4.0 Hz, NH_3_-21), 7.85 (1H, t, *J* = 5.8 Hz, NH-17), 7.50 (1H, d, *J* = 8.0 Hz, H-9), 7.36 (1H, d, *J* = 8.0 Hz, H-6), 7.20 (1H, d, *J* = 2.4 Hz, H-4), 7.08 (1H, ddd, *J* = 8.5, 8.0, 1.0 Hz, H-7), 7.00 (1H, ddd, *J* = 8.5, 8.0, 1.0 Hz, H-8), 4.61 (1H, dt, *J* = 8.8, 6.5 Hz, H-1), 3.89–3.80 (1H, m, H-13), 3.60 (3H, br s, OMe), 3.24–3.17 (1H, m, H-2a), 3.16–3.07 (3H, m, H-2b, H_2_-16), 1.78–1.69 (2H, m, H_2_-14), 1.59–1.48 (2H, m, H_2_-15); ^13^C NMR (100 MHz, DMSO-*d*_6_) δ 171.8 (C-10), 168.8 (C-12), 156.9 (C-18), 136.2 (C-5a), 127.0 (C-9a), 124.0 (C-4), 121.1 (C-7), 118.6 (C-8), 117.9 (C-9), 111.6 (C-6), 108.9 (C-3), 53.4 (C-1), 52.0 (OMe), 51.7 (C-13), 40.3 (C-16), 28.5 (C-14), 27.0 (C-2), 24.0 (C-15); (+)-HRESIMS [M + H]^+^ *m*/*z* 375.2124 (calcd for C_18_H_27_N_6_O_3_, 375.2139).

#### 3.1.9. Methyl d-arginyl-d-tryptophanate bis(2,2,2-trifluoroacetate) (**4b**)

Following general procedure B, Boc-protected dipeptide **3b** (52 mg, 0.110 mmol) was reacted with TFA (0.2 mL) in CH_2_Cl_2_ (2 mL) to afford the di-TFA salt of dipeptide **4b** as a pale yellow oil/gum (42 mg, 69%). [α]^19^_D_ −1.2 (*c* 0.424, MeOH); R*_f_* = 0.35 (MeOH); IR (ATR) *v*_max_ 3358, 3200, 3072, 2956, 2879, 1671, 1638, 1545, 1202, 1136 cm^−1^; ^1^H NMR (400 MHz, DMSO-*d*_6_) δ 10.94 (1H, d, *J* = 2.4 Hz, NH-5), 8.93 (1H, d, *J* = 7.1 Hz, NH-11), 8.18 (3H, d, *J* = 5.0 Hz, NH_3_-21), 7.80–7.74 (1H, m, NH-17), 7.49 (1H, d, *J* = 8.0 Hz, H-9), 7.36 (1H, d, *J* = 8.0 Hz, H-6), 7.20 (1H, d, *J* = 2.4 Hz, H-4), 7.11–7.05 (1H, m, H-7), 7.03–6.98 (1H, m, H-8), 4.64–4.57 (1H, m, H-1), 3.87–3.78 (1H, m, H-13), 3.60 (3H, br s, OMe), 3.23–3.16 (1H, m, H-2a), 3.15–3.07 (3H, m, H-2b, H_2_-16), 1.77–1.67 (2H, m, H_2_-14), 1.58–1.48 (2H, m, H_2_-15); ^13^C NMR (100 MHz, DMSO-*d*_6_) δ 171.7 (C-10), 168.7 (C-12), 156.8 (C-18), 136.2 (C-5a), 126.9 (C-9a), 123.9 (C-4), 121.1 (C-7), 118.5 (C-8), 117.9 (C-9), 111.5 (C-6), 108.8 (C-3), 53.3 (C-1), 52.0 (OMe), 51.6 (C-13), 40.2 (C-16), 28.4 (C-14), 27.0 (C-2), 23.9 (C-15); (+)-HRESIMS [M + H]^+^ *m*/*z* 375.2133 (calcd for C_18_H_27_N_6_O_3_, 375.2139).

#### 3.1.10. Methyl d-arginyl-l-tryptophanate bis(2,2,2-trifluoroacetate) (**4c**)

Following general procedure **B**, Boc-protected dipeptide **3c** (48 mg, 0.094 mmol) was reacted with TFA in CH_2_Cl_2_ to afford the di-TFA salt of dipeptide **4c** as a pale yellow oil/gum (31 mg, 55%). [α]^21^_D_ -11.2 (*c* 0.143, MeOH); R*_f_* = 0.09 (MeOH); IR (ATR) *v*_max_ 3350, 3197, 3077, 2961, 1663, 1624, 1556, 1435, 1356, 1182, 1131 cm^−1^; ^1^H NMR (400 MHz, DMSO-*d*_6_) δ 10.90–10.86 (1H, m, NH-5), 8.95 (1H, d, *J* = 8.0 Hz, NH-11), 8.20–8.11 (3H, m, NH_3_-21), 7.78–7.71 (1H, m, NH-17), 7.50 (1H, d, *J* = 7.8 Hz, H-9), 7.35 (1H, d, *J* = 7.8 Hz, H-6), 7.17–7.15 (1H, m, H-4), 7.10–7.04 (1H, m, H-7), 7.02–7.00 (1H, m, H-8), 4.68–4.61 (1H, m, H-1), 3.86–3.80 (1H, m, H-13), 3.62 (3H, br s, OMe), 3.24–3.17 (1H, m, H-2a), 3.11–3.02 (1H, m, H-2b), 3.01–2.92 (2H, m, H_2_-16), 1.62–1.49 (2H, m, H_2_-15), 1.36–1.25 (1H, m, H-14a), 1.25–1.12 (1H, m, 14b); ^13^C NMR (100 MHz, DMSO-*d*_6_) δ 171.8 (C-10), 168.4 (C-12), 156.8 (C-18), 136.1 (C-5a), 126.9 (C-9a), 124.0 (C-4), 121.1 (C-7), 118.5 (C-8), 117.9 (C-9), 111.5 (C-6), 109.0 (C-3), 53.0 (C-1), 52.1 (OMe), 51.6 (C-13), 40.0 (C-16), 28.3 (C-15), 27.4 (C-2), 23.7 (C-14); (+)-HRESIMS [M + H]^+^ *m*/*z* 375.2139 (calcd for C_18_H_27_N_6_O_3_, 375.2139).

#### 3.1.11. Methyl l-arginyl-d-tryptophanate bis(2,2,2-trifluoroacetate) (**4d**)

Following general procedure B, Boc-protected dipeptide **3d** (35 mg, 0.068 mmol) was reacted with TFA in CH_2_Cl_2_ to afford the di-TFA salt of dipeptide **4d** as a pale-yellow oil/gum (23 mg, 56%). [α]^19^_D_ +27 (*c* 0.10, MeOH); R*_f_* = 0.35 (MeOH); IR (ATR) *v*_max_ 3347, 3195, 3080, 2948, 2871, 1666, 1624, 1590, 1439, 1200, 1184, 1134 cm^−1^; ^1^H NMR (400 MHz, DMSO-*d*_6_) δ 10.91–10.87 (1H, m, NH-5), 8.95 (1H, d, *J* = 7.8 Hz, NH-11), 7.86–7.23 (1H, m, NH-17), 7.50 (1H, d, *J* = 7.8 Hz, H-9), 7.35 (1H, d, *J* = 7.8 Hz, H-6), 7.18–7.13 (1H, m, H-4), 7.11–7.04 (1H, m, H-7), 7.03–6.97 (1H, m, H-8), 4.64 (1H, dt, *J* = 9.2, 6.7 Hz, H-1), 3.86–3.77 (1H, m, H-13), 3.62 (3H, br s, OMe), 3.25–3.17 (1H, m, H-2a), 3.11–3.02 (1H, m, H-2b), 3.02–2.91 (2H, m, H_2_-16), 1.65–1.49 (2H, m, H_2_-15), 1.37–1.25 (1H, m, H-14a), 1.25–1.14 (1H, m, H-14b); ^13^C NMR (100 MHz, DMSO-*d*_6_) δ 171.8 (C-10), 168.4 (C-12), 156.9 (C-18), 136.1 (C-5a), 126.9 (C-9a), 124.0 (C-4), 121.1 (C-7), 118.5 (C-8), 117.9 (C-9), 111.5 (C-6), 108.9 (C-3), 53.0 (C-1), 52.0 (OMe), 51.7 (C-13), 40.0 (C-16), 28.3 (C-15), 27.4 (C-2), 23.7 (C-14); (+)-HRESIMS [M + H]^+^ *m*/*z* 375.2134 (calcd for C_18_H_27_N_6_O_3_, 375.2139).

#### 3.1.12. Cyclo(l-Trp-l-Arg) 2,2,2-trifluoroacetate (**5a**)

Following general procedure C, dipeptide **4a** (113 mg, 0.189 mmol) was reacted with NH_4_OH (0.13 mL) in MeOH (0.76 mL) to afford the TFA salt of cyclo(l-Trp-l-Arg) (**5a**) as a pale-yellow oil/gum (47 mg, 55%). [α]^24^_D_ -10.5 (*c* 0.105, MeOH); ECD (*c* 0.00035, MeOH) λ (Δε) 194 (0), 210 (−17.4), 223 (0), 231 (+10.2); R*_f_* = 0.60 (MeOH); IR (ATR) *v*_max_ 3363, 3233, 2969, 1659, 1648, 1457, 1137, 1106, 748 cm^−1^; ^1^H NMR (400 MHz, DMSO-*d*_6_) δ 10.88–10.84 (1H, m, NH-17), 8.09 (1H, d, *J* = 2.0 Hz, NH-4), 8.01–7.97 (1H, m, NH-1), 7.57 (1H, d, *J* = 7.9 Hz, H-21), 7.33 (1H, d, *J* = 7.9 Hz, H-18), 7.27–7.19 (1H, m, NH-10), 7.08–7.04 (1H, m, H-16), 7.05–7.00 (1H, m, H-19), 7.00–6.91 (1H, m, H-20), 4.14–4.09 (1H, br m, H-3), 3.59–3.53 (1H, br m, H-6), 3.23 (1H, dd, *J* = 14.5, 4.5 Hz, H-14a), 3.04 (1H, dd, *J* = 14.5, 4.5 Hz, H-14b), 2.72–2.64 (2H, m, H_2_-9), 1.08–0.95 (1H, m, H-7a), 0.95–0.78 (2H, m, H_2_-8), 0.67–0.55 (1H, m, H-7b); ^13^C NMR (100 MHz, DMSO-*d*_6_) δ 167.2 (C-2), 166.9 (C-5), 156.7 (C-11), 135.9 (C-17a), 127.8 (C-21a), 124.6 (C-16), 120.8 (C-19), 119.0 (C-21), 118.4 (C-20), 111.2 (C-18), 108.6 (C-15), 55.5 (C-3), 53.4 (C-6), 40.1 (C-9), 30.6 (C-7), 29.0 (C-14), 23.4 (C-8); (+)-HRESIMS [M + H]^+^ *m*/*z* 343.1874 (calcd for C_17_H_23_N_6_O_2_, 343.1877).

^1^H NMR (400 MHz, CD_3_OD) δ 7.63 (1H, d, *J* = 8.0 Hz, H-21), 7.35 (1H, d, *J* = 8.0 Hz, H-18), 7.12–7.07 (1H, m, H-19), 7.08 (1H, br s, H-16), 7.04–6.98 (1H, m, H-20), 4.31 (1H, ddd, *J* = 4.6, 4.0, 1.2 Hz, H-3), 3.68 (1H, ddd, *J* = 7.4, 6.5, 1.2 Hz, H-6), 3.49 (1H, dd, *J* = 14.6, 3.7 Hz, H-14a), 3.14 (1H, dd, *J* = 14.6, 4.6 Hz, H-14b), 2.62 (2H, t, *J* = 7.1 Hz, H_2_-9), 0.93–0.80 (2H, m, H-7a, H-8a), 0.77–0.65 (1H, m, H-8b), 0.56–0.45 (1H, m, H-7b); ^13^C NMR (100 MHz, CD_3_OD) δ 169.9 (C-2), 169.5 (C-5), 158.5 (C-11), 137.8 (C-17a), 129.4 (C-21a), 126.1 (C-16), 122.5 (C-19), 120.3 (C-20), 120.2 (C-21), 112.2 (C-18), 109.6 (C-15), 57.5 (C-3), 55.2 (C-6), 41.7 (C-9), 32.1 (C-7), 30.5 (C-14), 24.5 (C-8).

#### 3.1.13. Cyclo(d-Trp-d-Arg) 2,2,2-trifluoroacetate (**5b**)

Following general procedure C, dipeptide **4b** (27 mg, 0.045 mmol) was reacted with NH_4_OH (0.03 mL) in MeOH (0.18 mL) to afford the TFA salt of cyclo(d-trp-d-Arg) (**5b**) as a pale-yellow oil/gum (15 mg, 71%). [α]^24^_D_ +10.7 (*c* 0.103, MeOH); ECD (*c* 0.00037, MeOH) λ (Δε) 195 (0), 213 (+18.7), 223 (0), 230 (-9.83); R*_f_* = 0.60 (MeOH); IR (ATR) *v*_max_ 3350, 3216, 2979, 1659, 1654, 1457, 1201, 1137 cm^−1^; ^1^H NMR (400 MHz, DMSO-*d*_6_) δ 10.87–10.83 (1H, m, NH-17), 8.09 (1H, d, *J* = 2.2 Hz, NH-4), 8.00 (1H, d, *J* = 2.2 Hz, NH-1), 7.57 (1H, d, *J* = 8.0 Hz, H-21), 7.32 (1H, d, *J* = 8.0 Hz, H-18), 7.25–7.12 (1H, m, NH-10), 7.07–7.05 (1H, m, H-16), 7.06–7.01 (1H, m, H-19), 6.97–6.91 (1H, m, H-20), 4.14–4.09 (1H, br m, H-3), 3.59–3.53 (1H, br m, H-6), 3.23 (1H, dd, *J* = 14.6, 4.4 Hz, H-14a), 3.04 (1H, dd, *J* = 14.6, 4.6 Hz, H-14b), 2.74–2.64 (2H, m, H_2_-9), 1.08–0.96 (1H, m, H-7a), 0.96–0.79 (2H, m, H_2_-8), 0.66–0.54 (1H, m, H-7b); ^13^C NMR (100 MHz, DMSO-*d*_6_) δ 167.2 (C-2), 166.9 (C-5), 156.6 (C-11), 135.9 (C-17a), 127.8 (C-21a), 124.6 (C-16), 120.8 (C-19), 119.0 (C-21), 118.4 (C-20), 111.1 (C-18), 108.7 (C-15), 55.4 (C-3), 53.4 (C-6), 40.1 (C-9), 30.6 (C-7), 29.0 (C-14), 23.4 (C-8); (+)-HRESIMS [M + H]^+^ *m*/*z* 343.1867 (calcd for C_17_H_23_N_6_O_2_, 343.1877).

^1^H NMR (400 MHz, CD_3_OD) δ 7.63 (1H, d, *J* = 8.0 Hz, H-21), 7.35 (1H, d, *J* = 8.0 Hz, H-18), 7.09 (1H, ddd, *J* = 8.2, 8.0, 1.0 Hz, H-19), 7.08 (1H, br s, H-16), 7.01 (1H, ddd, *J* = 8.2, 8.0, 1.0 Hz, H-20), 4.31 (1H, ddd, *J* = 5.0, 4.0, 1.0 Hz, H-3), 3.68 (1H, ddd, *J* = 7.5, 6.2, 1.2 Hz, H-6), 3.48 (1H, dd, *J* = 14.6, 3.8 Hz, H-14a), 3.14 (1H dd, *J* = 14.6, 4.6 Hz, H-14b), 2.62 (2H, t, *J* = 7.1 Hz, H_2_-9), 0.94–0.79 (2H, m, H-7a, H-8a), 0.77–0.64 (1H, m, H-8b), 0.54–0.43 (1H, m, H-7b); ^13^C NMR (100 MHz, CD_3_OD) δ 169.9 (C-2), 169.5 (C-5), 158.4 (C-11), 137.8 (C-17a), 129.4 (C-21a), 126.0 (C-16), 122.5 (C-19), 120.2 (C-20, C-21), 112.2 (C-18), 109.6 (C-15), 57.5 (C-3), 55.2 (C-6), 41.7 (C-9), 32.0 (C-7), 30.4 (C-14), 24.5 (C-8).

#### 3.1.14. Cyclo(l-Trp-d-Arg) 2,2,2-trifluoroacetate (**5c**)

Following general procedure C, dipeptide **4c** (118.1 mg, 0.196 mmol) was reacted with NH_4_OH (0.13 mL) in MeOH (0.78 mL) to afford the TFA salt of cyclo(l-Trp-d-Arg) (**5c**) as a pale-yellow oil/gum (48 mg, 54%). [α]^21^_D_ +26.0 (*c* 0.131, MeOH); ECD (*c* 0.00035, MeOH) λ (Δε) 204 (0), 216 (−10.6), 227 (0), 235 (+2.95); R*_f_* = 0.49 (MeOH); IR (ATR) *v*_max_ 3356, 3215, 2961, 2903, 1662, 1458, 1202, 1138 cm^−1^; ^1^H NMR (400 MHz, DMSO-*d*_6_) δ 10.91 (1H, d, *J* = 2.0 Hz, NH-17), 8.10 (1H, d, *J* = 2.2 Hz, NH-4), 7.91–7.90 (1H, m, NH-1), 7.57 (1H, d, *J* = 7.9 Hz, H-21), 7.47 (1H, t, *J* = 5.2 Hz, NH-10), 7.32 (1H, d, *J* = 7.9 Hz, H-18), 7.07 (1H, d, *J* = 2.0 Hz, H-16), 7.04 (1H, ddd, *J* = 8.4, 7.9, 1.0 Hz, H-19), 6.95 (1H, ddd, *J* = 8.4, 7.9, 1.0 Hz, H-20), 4.10–4.05 (1H, br m, H-3), 3.29–3.22 (1H, dd, *J* = 14.5, 4.5 Hz, H-14a), 3.10–3.01 (1H, m, H-14b), 3.08–3.03 (1H, m, H-6), 3.02–3.00 (2H, m, H_2_-9), 1.61–1.50 (1H, m, H-7a), 1.50–1.42 (1H, m, H-7b), 1.42–1.30 (2H, m, H_2_-8); ^13^C NMR (100 MHz, DMSO-*d*_6_) δ 168.2 (C-2), 167.5 (C-5), 156.7 (C-11), 135.9 (C-17a), 127.6 (C-21a), 124.6 (C-16), 120.9 (C-19), 118.8 (C-21), 118.4 (C-20), 111.2 (C-18), 108.4 (C-15), 55.4 (C-3), 52.9 (C-6), 40.4 (C-9), 29.1 (C-7), 28.9 (C-14), 23.5 (C-8); (+)-HRESIMS [M + H]^+^ *m*/*z* 343.1874 (calcd for C_17_H_23_N_6_O_2_, 343.1877).

^1^H NMR (400 MHz, CD_3_OD) δ 7.60 (1H, d, *J* = 8.0 Hz, H-21), 7.33 (1H, d, *J* = 8.0 Hz, H-18), 7.11–7.05 (1H, m, H-19), 7.06 (1H, br s, H-16), 7.00 (1H, ddd, *J* = 8.5, 8.0, 1.0 Hz, H-20), 4.22 (1H, t, *J* = 4.0 Hz, H-3), 3.46 (1H, dd, *J* = 14.6, 4.0 Hz, H-14a), 3.15 (1H, dd, *J* = 14.6, 4.4 Hz, H-14b), 3.04 (2H, t, *J* = 6.8 Hz, H_2_-9), 2.77 (1H, t, *J* = 4.0 Hz, H-6), 1.73–1.62 (1H, m, H-7a), 1.54–1.32 (3H, m, H-7b, H_2_-8); ^13^C NMR (100 MHz, CD_3_OD) δ 171.4 (C-2), 170.3 (C-5), 158.6 (C-11), 137.9 (C-17a), 128.8 (C-21a), 126.1 (C-16), 122.6 (C-19), 120.2 (C-20), 119.8 (C-21), 112.2 (C-18), 109.0 (C-15), 57.7 (C-3), 54.3 (C-6), 42.0 (C-9), 31.1 (C-14), 29.6 (C-7), 24.3 (C-8).

#### 3.1.15. Cyclo(d-Trp-l-Arg) 2,2,2-trifluoroacetate (**5d**)

Following general procedure C, dipeptide **4d** (52 mg, 0.086 mmol) was reacted with NH_4_OH (0.057 mL) in MeOH (0.344 mL) to afford the TFA salt of cyclo(d-Trp-l-Arg) (**5d**) as a pale-yellow gum (18 mg, 47%). [α]^19^_D_ -26.4, (*c* 0.421, MeOH); ECD (*c* 0.00035, MeOH) λ (Δε) 204 (0), 216 (+11.8), 227 (0), 234 (-1.75); R*_f_* = 0.49 (MeOH); IR (ATR) *v*_max_ 3342, 3214, 2964, 1662, 1651, 1456, 1431, 1201, 1135 cm^−1^; ^1^H NMR (400 MHz, DMSO-*d*_6_) δ 10.93 (1H, d, *J* = 2.0 Hz, NH-17), 8.11–8.09 (1H, m, NH-4), 7.92–7.90 (1H, m, NH-1), 7.63 (1H, t, *J* = 5.5 Hz, NH-10), 7.57 (1H, d, *J* = 8.1 Hz, H-21), 7.32 (1H, d, *J* = 8.1 Hz, H-18), 7.07 (1H, d, *J* = 2.0 Hz, H-16), 7.04 (1H, ddd, *J* = 8.1, 7.5, 1.0 Hz, H-19), 6.94 (1H, ddd, *J* = 8.1, 7.5, 1.0 Hz, H-20), 4.10–4.04 (1H, br m, H-3), 3.29–3.21 (1H, dd, *J* = 14.5, 4.4 Hz, H-14a), 3.09–3.03 (1H, m, H-6), 3.08–3.01 (1H, m, H-14b), 3.02–3.00 (2H, m, H_2_-9), 1.61–1.45 (2H, m, H_2_-7), 1.43–1.31 (2H, m, H_2_-8); ^13^C NMR (100 MHz, DMSO-*d*_6_) δ 168.2 (C-2), 167.5 (C-5), 156.8 (C-11), 135.9 (C-17a), 127.6 (C-21a), 124.6 (C-16), 120.9 (C-19), 118.8 (C-21), 118.4 (C-20), 111.2 (C-18), 108.4 (C-15), 55.4 (C-3), 52.9 (C-6), 40.5 (C-9), 29.1 (C-7), 28.9 (C-14), 23.5 (C-8); (+)-HRESIMS [M + H]^+^ *m*/*z* 343.1870 (calcd for C_17_H_23_N_6_O_2_, 343.1877).

^1^H NMR (400 MHz, CD_3_OD) δ 7.60 (1H, d, *J* = 8.0 Hz, H-21), 7.33 (1H, d, *J* = 8.0 Hz, H-18), 7.11–7.05 (1H, m, H-19), 7.06 (1H, br s, H-16), 7.03–6.98 (1H, m, H-20), 4.23 (1H, t, *J* = 4.1 Hz, H-3), 3.46 (1H, dd, *J* = 14.6, 4.0 Hz, H-14a), 3.15 (1H, dd, *J* = 14.6, 4.5 Hz, H-14b), 3.04 (2H, t, *J* = 6.7 Hz, H-9), 2.78 (1H, t, *J* = 3.9 Hz, H-6), 1.73–1.61 (1H, m, H-7a), 1.54–1.33 (3H, m, H-7b, H_2_-8); ^13^C NMR (100 MHz, CD_3_OD) δ 171.4 (C-2), 170.3 (C-5), 158.6 (C-11), 137.9 (C-17a), 128.8 (C-21a), 126.1 (C-16), 122.6 (C-19), 120.2 (C-20), 119.8 (C-21), 112.2 (C-18), 109.0 (C-15), 57.7 (C-3), 54.3 (C-6), 42.0 (C-9), 31.1 (C-14), 29.6 (C-7), 24.3 (C-8).

### 3.2. Antimicrobial Assays

The susceptibility of bacterial strains *S. aureus* (ATCC 25923 or 29213) and *P. aeruginosa* (ATCC 27853 or PAO1) to antibiotics and compounds was determined in microplates using the standard broth dilution method in accordance with the recommendations of the Comité de l’AntibioGramme de la Société Française de Microbiologie (CA-SFM). Briefly, the minimal inhibitory concentrations (MICs) were determined with an inoculum of 10^5^ CFU in 200 µL of Mueller–Hinton broth (MHB) containing two-fold serial dilutions of each drug. The MIC was defined as the lowest concentration of drug that completely inhibited visible growth after incubation for 18 h at 37 °C. To determine all MICs, the measurements were independently repeated in triplicate.

Additional antimicrobial evaluation against *S. aureus* (MRSA) (ATCC 43300), *E. coli* (ATCC 25922), *K. pneumoniae* (ATCC 700603), *A. baumannii* (ATCC 19606), *C. albicans* (ATCC 90028), and *C. neoformans* (ATCC 208821) was undertaken at the Community for Open Antimicrobial Drug Discovery at The University of Queensland (Australia) according to their standard protocols [3]. For antimicrobial assays, the tested strains were cultured in either Luria broth (LB) (In Vitro Technologies, USB75852, Victoria, Australia), nutrient broth (NB) (Becton Dickson, 234000, New South Wales, Australia), or MHB at 37 °C overnight. A sample of culture was then diluted 40-fold in fresh MHB and incubated at 37 °C for 1.5−2 h. The compounds were serially diluted 2-fold across the wells of 96-well plates (Corning 3641, nonbinding surface), with compound concentrations ranging from 0.015 to 64 μg/mL, plated in duplicate. The resultant mid log phase cultures were diluted to the final concentration of 1 × 10^6^ CFU/mL; then, 50 μL was added to each well of the compound-containing plates, yielding a final compound concentration range of 0.008 to 32 μg/mL and a cell density of 5 × 10^5^ CFU/mL. All plates were then covered and incubated at 37 °C for 18 h. Resazurin was added at 0.001% final concentration to each well and incubated for 2 h before MICs were read by eye.

For the antifungal assay, fungi strains were cultured for 3 days on YPD agar at 30 °C. A yeast suspension of 1 × 10^6^ to 5 × 10^6^ CFU/mL was prepared from five colonies. These stock suspensions were diluted with yeast nitrogen base (YNB) (Becton Dickinson, 233520, New South Wales, Australia) broth to a final concentration of 2.5 × 10^3^ CFU/mL. The compounds were serially diluted 2-fold across the wells of 96-well plates (Corning 3641, nonbinding surface), with compound concentrations ranging from 0.015 to 64 μg/mL and final volumes of 50 μL, plated in duplicate. Then, 50 μL of the fungi suspension that was previously prepared in YNB broth to the final concentration of 2.5 × 10^3^ CFU/mL was added to each well of the compound-containing plates, yielding a final compound concentration range of 0.008 to 32 μg/mL. Plates were covered and incubated at 35 °C for 36 h without shaking. *C. albicans* MICs were determined by measuring the absorbance at OD_530_. For *C. neoformans*, resazurin was added at 0.006% final concentration to each well and incubated for a further 3 h before MICs were determined by measuring the absorbance at OD_570−600_.

Colistin and vancomycin were used as positive bacterial inhibitor standards for Gram-negative and Gram-positive bacteria, respectively. Fluconazole was used as a positive fungal inhibitor standard for *C. albicans* and *C. neoformans*. The antibiotics were provided in 4 concentrations, with 2 above and 2 below its MIC value, and plated into the first 8 wells of column 23 of the 384-well NBS plates. The quality control (QC) of the assays was determined by the antimicrobial controls and the Z’-factor (using positive and negative controls). Each plate was deemed to fulfil the quality criteria (pass QC) if the Z’-factor was above 0.4, and the antimicrobial standards showed a full range of activity, with full growth inhibition at their highest concentration and no growth inhibition at their lowest concentration.

### 3.3. Determination of the MICs of Antibiotics in the Presence of Synergizing Compounds

Briefly, restoring enhancer concentrations were determined with an inoculum of 5 × 10^5^ CFU in 200 µL of MHB containing two-fold serial dilutions of each derivative in the presence of either doxycycline or ampicillin at 2 µg/mL. The lowest concentration of the synthetic material that completely inhibited visible growth after incubation for 18 h at 37 °C was determined. These measurements were independently repeated in triplicate.

### 3.4. Cytotoxicity Assays

HEK293 cells (ATCC CRL-1573) were counted manually in a Neubauer hemocytometer and plated at a density of 5000 cells/well into each well of the 384-well plates containing the 25× (2 μL) concentrated compounds. The medium used was Dulbecco’s modified eagle medium (DMEM) supplemented with 10% fetal bovine serum (FBS). Cells were incubated together with the compounds for 20 h at 37 °C, 5% CO_2_. To measure cytotoxicity, 5 μL (equals 100 μM final) of resazurin was added to each well after incubation and incubated for further 3 h at 37 °C with 5% CO_2_. After the final incubation, fluorescence intensity was measured as Fex 560/10 nm, em 590/10 nm (F_560/590_) using a Tecan M1000 Pro monochromator plate reader. CC_50_ values (concentration at 50% cytotoxicity) were calculated by normalizing the fluorescence readout, with 74 μg/mL tamoxifen as negative control (0%) and normal cell growth as positive control (100%). The concentration-dependent percentage cytotoxicity was fitted to a dose-response function (using Pipeline Pilot) and CC_50_ values were determined.

### 3.5. Hemolytic Assays

Human whole blood (ARCBS 5400 00150) was washed three times with 3 volumes of 0.9% NaCl and then resuspended in the same to a concentration of 0.5 × 10^8^ cells/mL, as determined by manual cell count in a Neubauer hemocytometer. The washed cells were then added to the 384-well compound-containing plates for a final volume of 50 μL. After a 10 min shake on a plate shaker the plates were then incubated for 1 h at 37 °C. After incubation, the plates were centrifuged at 1000× *g* for 10 min to pellet cells and debris, and 25 μL of the supernatant was then transferred to a polystyrene 384-well assay plate. Hemolysis was determined by measuring the supernatant absorbance at 405 mm (OD_405_). The absorbance was measured using a Tecan M1000 Pro monochromator plate reader. HC_10_ and HC_50_ (concentration at 10% and 50% hemolysis, respectively) were calculated by curve fitting the inhibition values vs. log (concentration) using a sigmoidal dose-response function with variable fitting values for top, bottom, and slope.

## 4. Conclusions

In summary, we synthesized the four diastereomers of diketopiperazine cyclo(Trp-Arg) and characterized them extensively using NMR, ESIMS, and chiroptical methods. Our data, while being in close agreement with previously reported spectroscopic data [9,12], raise uncertainty regarding the structure of cyclo(l-Trp-l-Arg) CDP2 reported from the bacterium *Achromobacter* sp. [8]. Based upon available data, we are not able to assign a structure or relative configuration to natural product CDP3 **2** at this time. Antimicrobial and antibiotic enhancing activities attributed to the natural products [8] were not observed for the synthesized diketopiperazines, leading us to conclude that these specific cyclic dipeptides do not represent viable templates for the development of new treatments for microbial infections. This study also illustrates the key role total synthesis continues to play in the establishment/confirmation of the absolute configuration of natural products.

## Figures and Tables

**Table 1:** Specific rotation values observed for diketopiperazines **5a–d**.

Cmpd	[α]_D_^1^	Lit.
**5a** cyclo(l-Trp-l-Arg)	−10.5 (*c* 0.105, MeOH)	−13 [12]
**5b** cyclo(d-Trp-d-Arg)	+10.7 (*c* 0.103, MeOH)	
**5c** cyclo(l-Trp-d-Arg)	+26.0 (*c* 0.131, MeOH)	
**5d** cyclo(d-Trp-l-Arg)	−26.4 (*c* 0.421, MeOH)	

^1^ Acquired in MeOH at the concentration given in g/100 mL.

## Data Availability

Data are contained within the article or Supplementary Material.

## Supplementary Materials

The following supporting information can be downloaded at: https://www.mdpi.com/article/10.3390/molecules27185913/s1, Figure S1: ^1^H and ^13^C NMR data (DMSO-*d*_6_) for cyclo(l-Trp-l-Arg) (**5a**) Figure S2: ^1^H and ^13^C NMR data (CD_3_OD) for cyclo(l-Trp-l-Arg) (**5a**); Figure S3: ^1^H and ^13^C NMR data (DMSO-*d*_6_) for cyclo(d-Trp-d-Arg) (**5b**); Figure S4: ^1^H and ^13^C NMR data (CD_3_OD) for cyclo(d-Trp-d-Arg) (**5b**); Figure S5: ^1^H and ^13^C NMR data (DMSO-*d*_6_) for cyclo(l-Trp-d-Arg) (**5c**); Figure S6: ^1^H and ^13^C NMR data (CD_3_OD) for cyclo(l-Trp-d-Arg) (**5c**); Figure S7: ^1^H and ^13^C NMR data (DMSO-*d*_6_) for cyclo(d-Trp-l-Arg) (**5d**); Figure S8: ^1^H and ^13^C NMR data (CD_3_OD) for cyclo(d-Trp-l-Arg) (**5d**); Table S1: Comparison of ^1^H NMR chemical shifts observed for **5a** and **5c** with corresponding shifts reported by Li et al. and calculated values of mean absolute error (MAE); Table S2: Comparison of ^13^C NMR chemical shifts (CD_3_OD) observed for **5a** and **5c** with corresponding shifts reported by Li et al. and calculated values of mean absolute error (MAE); Table S3: Comparison of ^1^H NMR chemical shifts reported for CDP 2 (**1**) with corresponding shifts observed for **5a** (cyclo(l-Trp-l-Arg) in either DMSO-*d*_6_ or CD_3_OD (with exchangeables in DMSO-*d*_6_) and calculated values of mean absolute error (MAE); Table S4: Comparison of ^1^H NMR chemical shifts reported for CDP 3 (**2**) with corresponding shifts observed for **5a** (cyclo(l-Trp-l-Arg) in either DMSO-*d*_6_ or CD_3_OD (with exchangeables in DMSO-*d*_6_) and calculated values of mean absolute error (MAE); Table S5: Comparison of ^13^C NMR chemical shifts reported for CDP 2 (**1**) with corresponding shifts observed for **5a** (cyclo(l-Trp-l-Arg) in either DMSO-*d*_6_ or CD_3_OD and calculated values of mean absolute error (MAE); Table S6: Comparison of ^13^C NMR chemical shifts reported for CDP 3 (**2**) with corresponding shifts observed for **5a** (cyclo(l-Trp-l-Arg) in either DMSO-*d*_6_ or CD_3_OD and calculated values of mean absolute error (MAE).

## Sample Availability

Samples of the final compounds **5a–d** are available from the authors.
