# Supplementary material for: Total Synthesis of the Four Stereoisomers of Cyclo(l-Trp-l-Arg) Raises Uncertainty of the Structures of the Natural Products and Invalidates Their Promising Antimicrobial Activities
Source: Molecules. 2022 Sep 12;27(18):5913. doi: 10.3390/molecules27185913 (PMC9501421; doi:10.3390/molecules27185913)
Supplement: Supplementary file 1 [file molecules-27-05913-s001.zip › molecules-1915220-supplementary.pdf]

Supplementary Material

Total synthesis of the four stereoisomers of cyclo(L-Trp-L-Arg) raises uncertainty of the structures of the natural products and invalidates their promising antimicrobial activities

Dan Chen [1], Daniel J. Park [1], Melissa M. Cadelis [1], Hana Douafer [2], Marie Lise Bourguet-Kondracki [3], Jean Michel Brunel [2], and Brent R. Copp [1,*]

1 School of Chemical Sciences, The University of Auckland, Waipapa Taumata Rau, Private Bag 92019, Auckland 1142, New Zealand
2 Aix-Marseille Universite, INSERM, SSA, MCT, Faculté de Pharmacie, 27 bd Jean Moulin, 13385 Marseille, France
3 Laboratoire Molécules de Communication et Adaptation des Micro-organismes, UMR 7245 CNRS, Muséum National d'Histoire Naturelle, 57 rue Cuvier (C.P. 54), 75005 Paris, France

Table of contents:

Figures S1 – S8, ^{1}H and ^{13}C NMR spectra (DMSO-d_6 and CD_3OD) for compounds **5a-d**.

Table S1. Comparison of ^{1}H NMR chemical shifts observed for **5a** and **5c** with corresponding shifts reported by Li *et al.* and calculated values of mean absolute error (MAE).

Table S2. Comparison of ^{13}C NMR chemical shifts (CD_3OD) observed for **5a** and **5c** with corresponding shifts reported by Li *et al.* and calculated values of mean absolute error (MAE).

Table S3. Comparison of ^{1}H NMR chemical shifts reported for CDP 2 (**1**) with corresponding shifts observed for **5a** (cyclo(L-Trp-L-Arg) in either DMSO-d_6 or CD_3OD (with exchangeables in DMSO-d_6) and calculated values of mean absolute error (MAE).

Table S4. Comparison of ^{1}H NMR chemical shifts reported for CDP 3 (**2**) with corresponding shifts observed for **5a** (cyclo(L-Trp-L-Arg) in either DMSO-d_6 or CD_3OD (with exchangeables in DMSO-d_6) and calculated values of mean absolute error (MAE).

Table S5. Comparison of ^{13}C NMR chemical shifts reported for CDP 2 (**1**) ("DMSO-d_6") with corresponding shifts observed for **5a** (cyclo(L-Trp-L-Arg) in either DMSO-d_6 or CD_3OD and calculated values of mean absolute error (MAE).

Table S6. Comparison of ^{13}C NMR chemical shifts reported for CDP 3 (**2**) with corresponding shifts observed for **5a** (cyclo(L-Trp-L-Arg) in either DMSO-d_6 or CD_3OD and calculated values of mean absolute error (MAE).

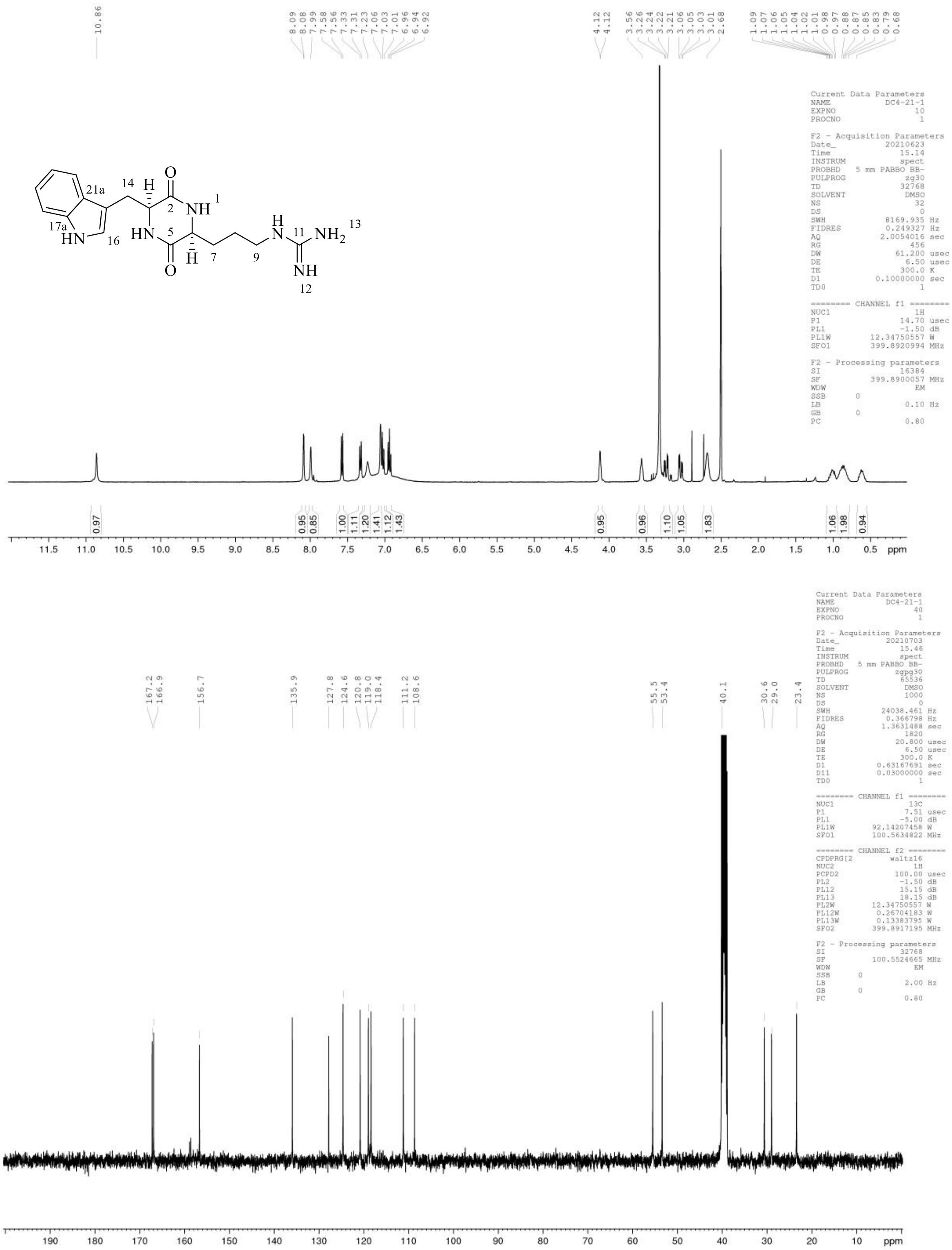

Figure S1. ^{1}H and ^{13}C NMR data (DMSO-d_6) for cyclo(L-Trp-L-Arg) (**5a**).

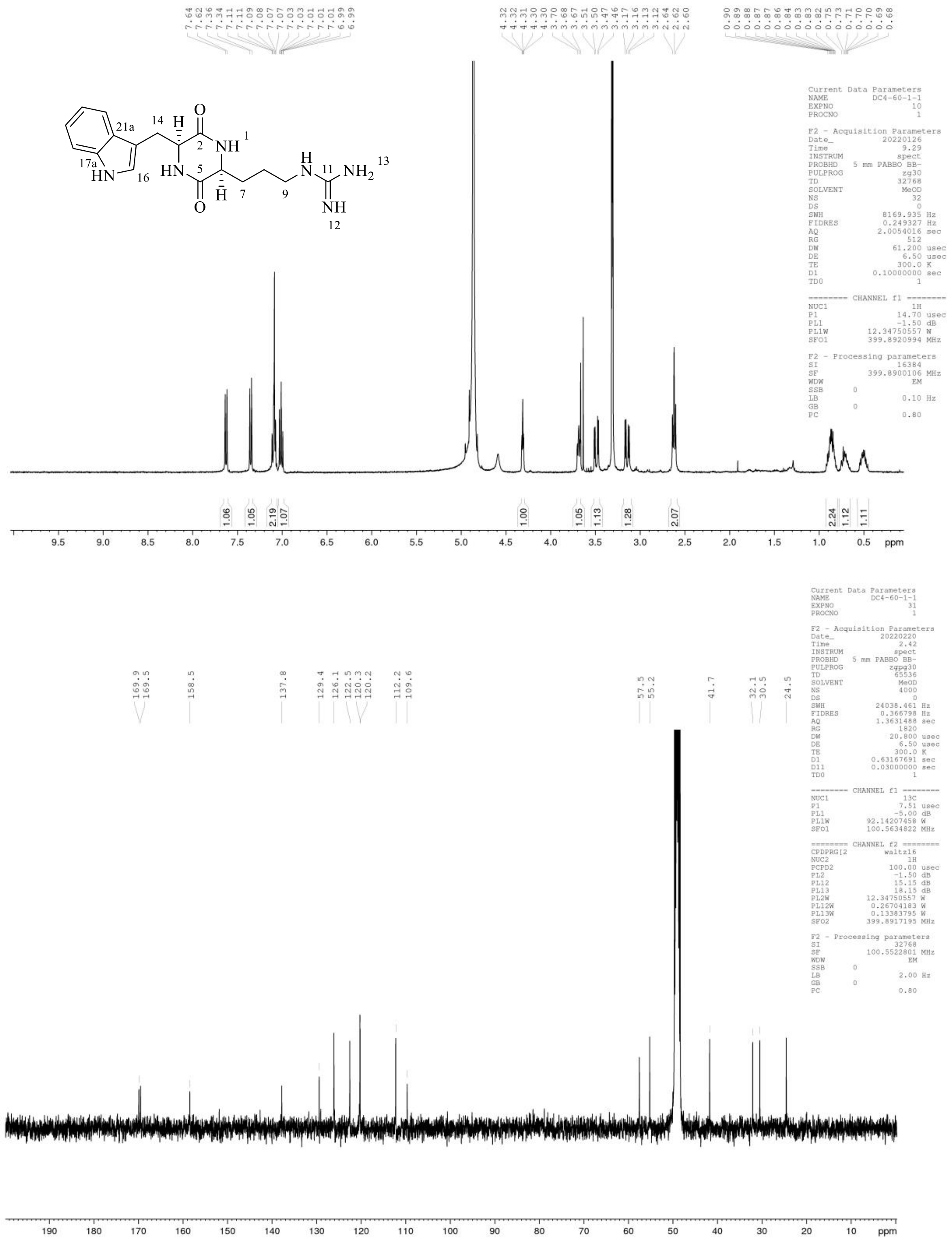

Figure S2. ^{1}H and ^{13}C NMR data (CD_3OD) for cyclo(L-Trp-L-Arg) (**5a**).

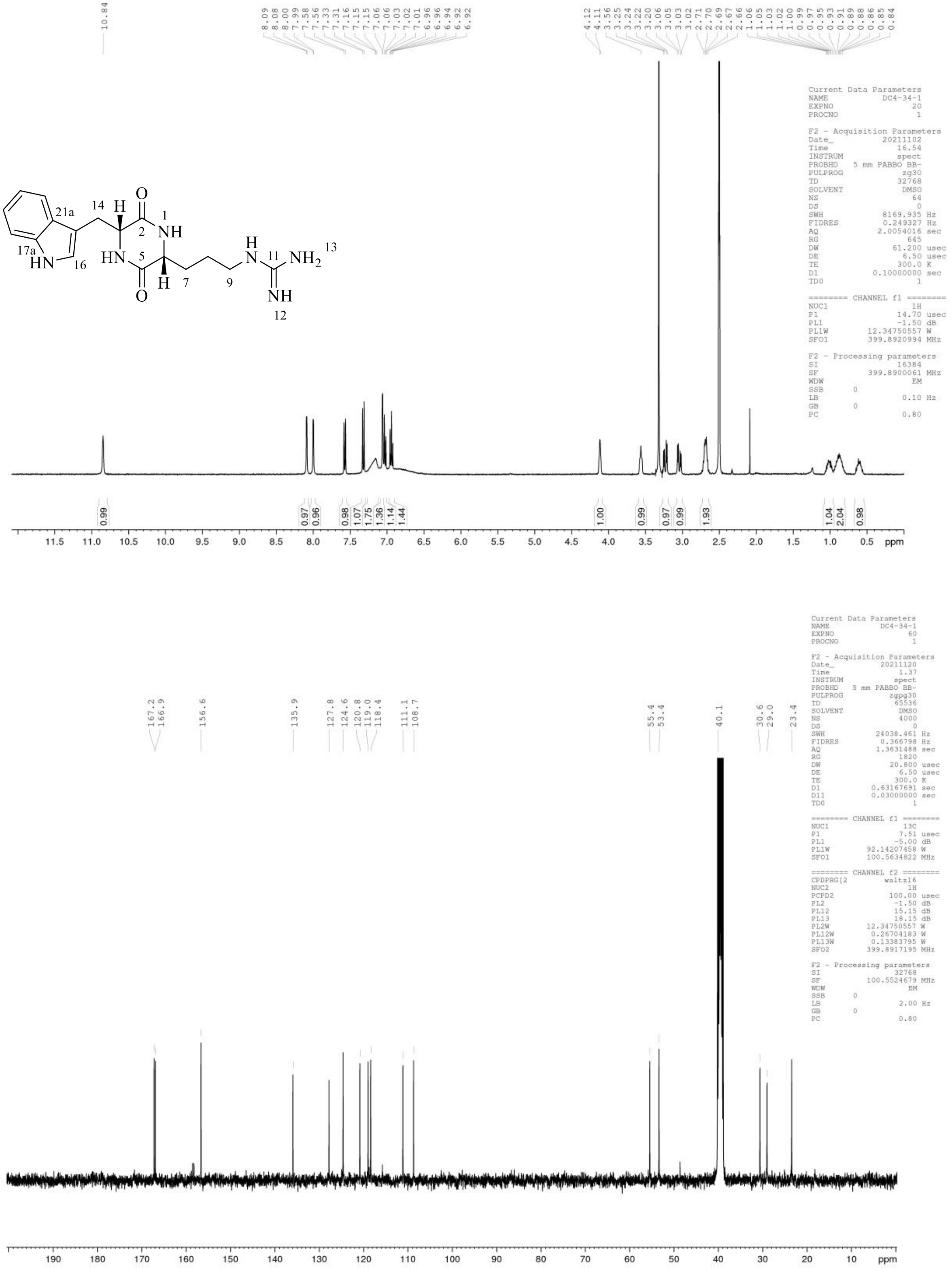

Figure S3. ^{1}H and ^{13}C NMR data (DMSO-d_6) for cyclo(D-Trp-D-Arg) (**5b**).

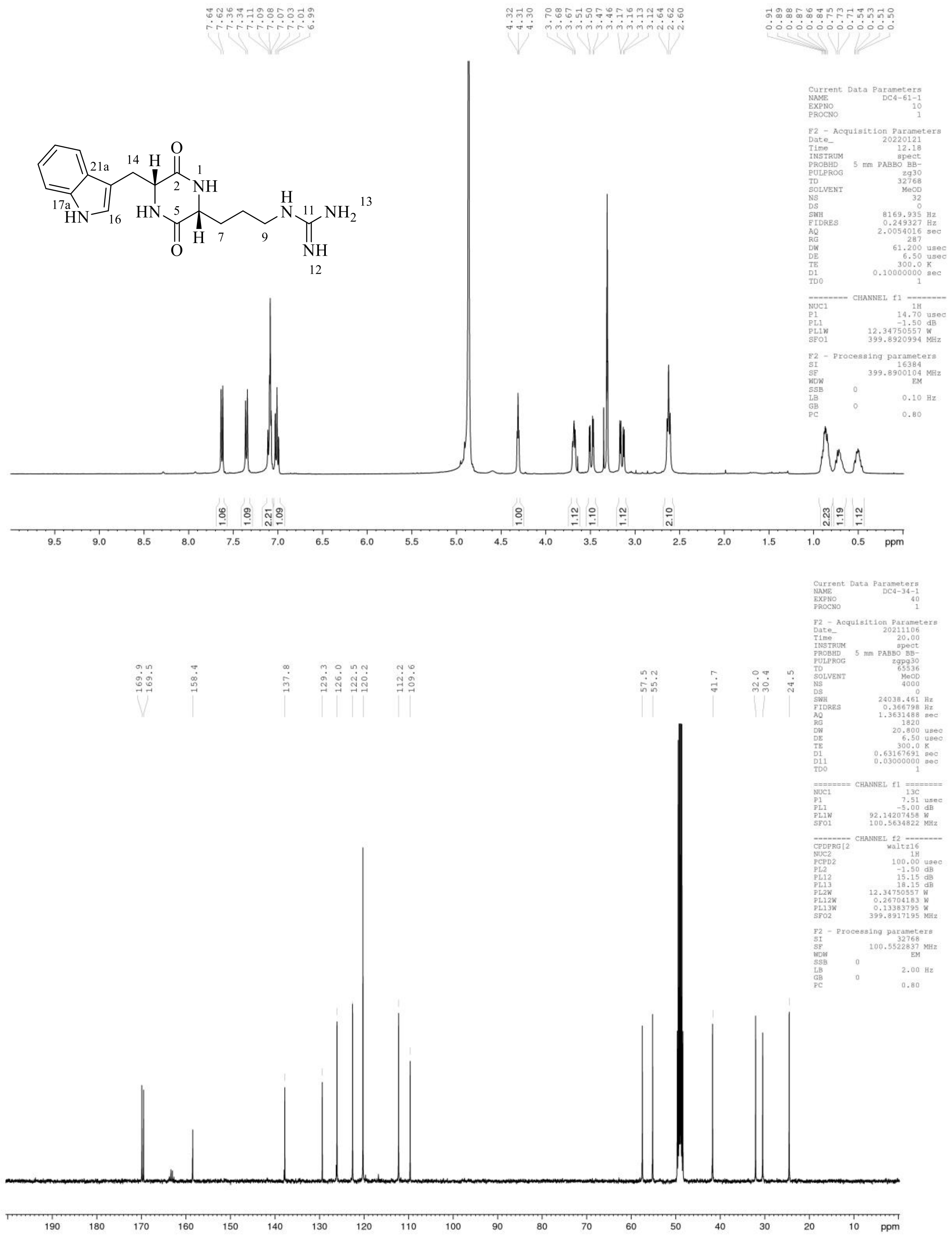

Figure S4. ^{1}H and ^{13}C NMR data (CD_3OD) for cyclo(D-Trp-D-Arg) (**5b**).

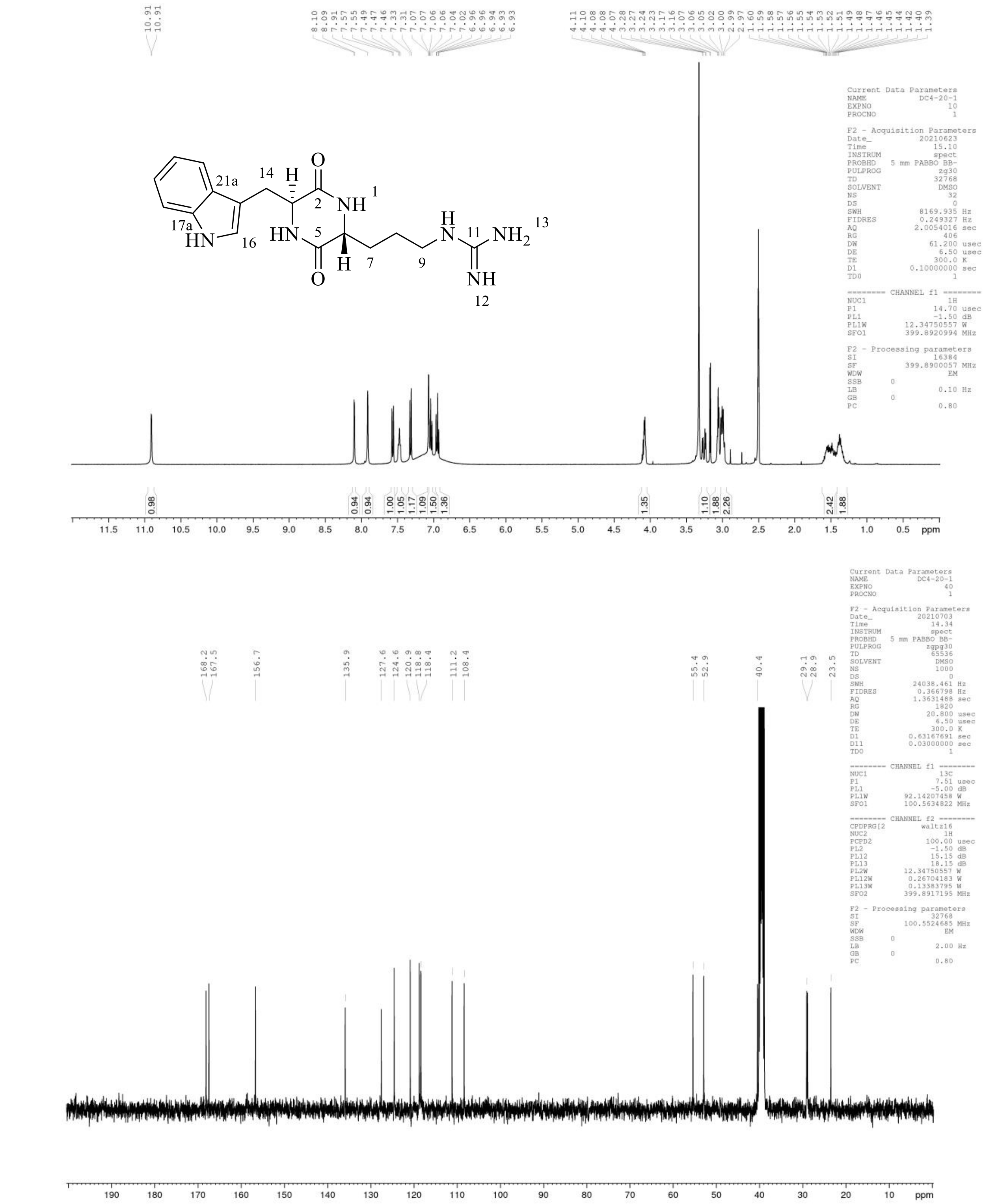

Figure S5. ^{1}H and ^{13}C NMR data (DMSO-d_6) for cyclo(L-Trp-D-Arg) (**5c**).

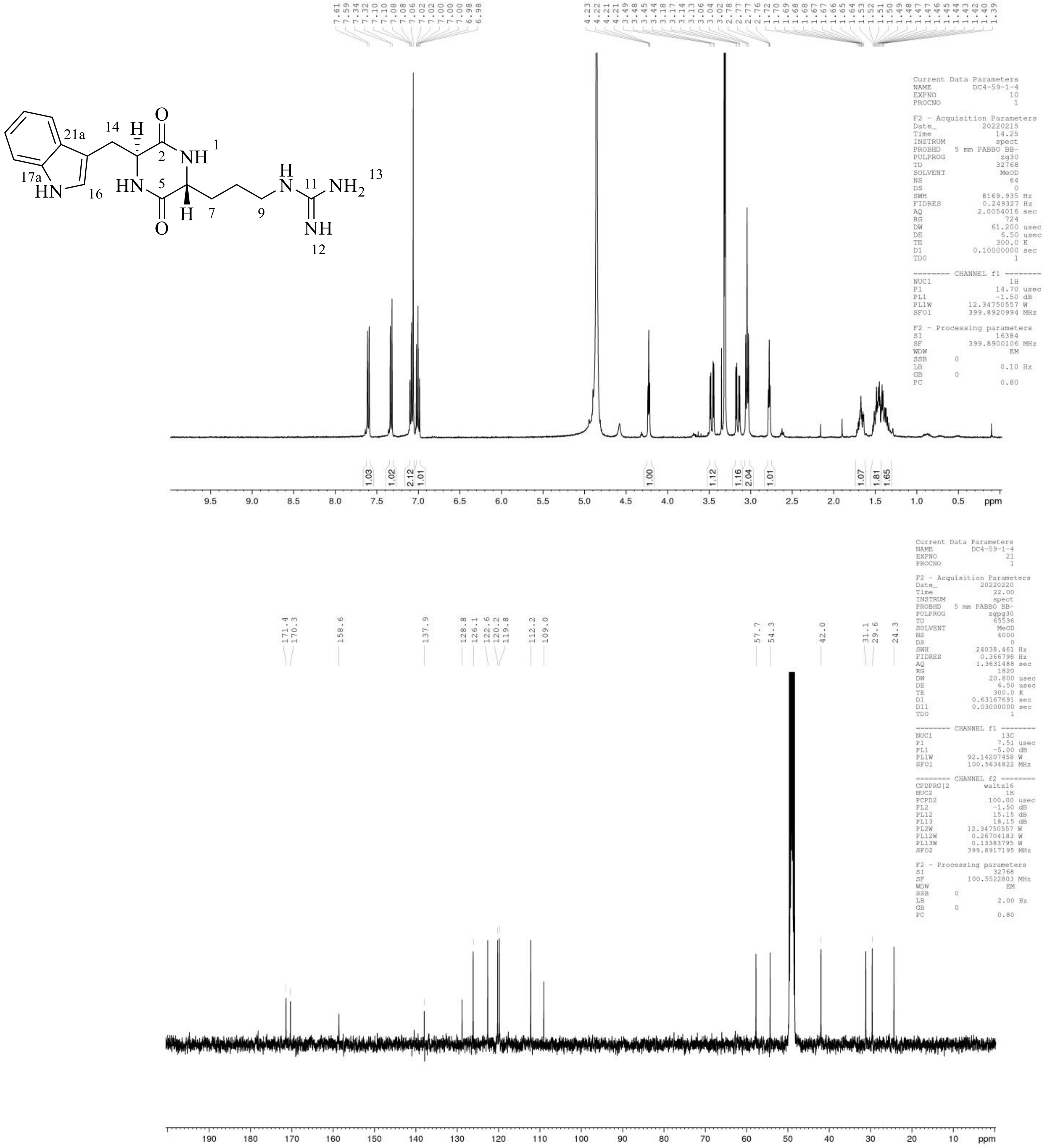

Figure S6. ^{1}H and ^{13}C NMR data (CD_3OD) for cyclo(L-Trp-D-Arg) (**5c**).

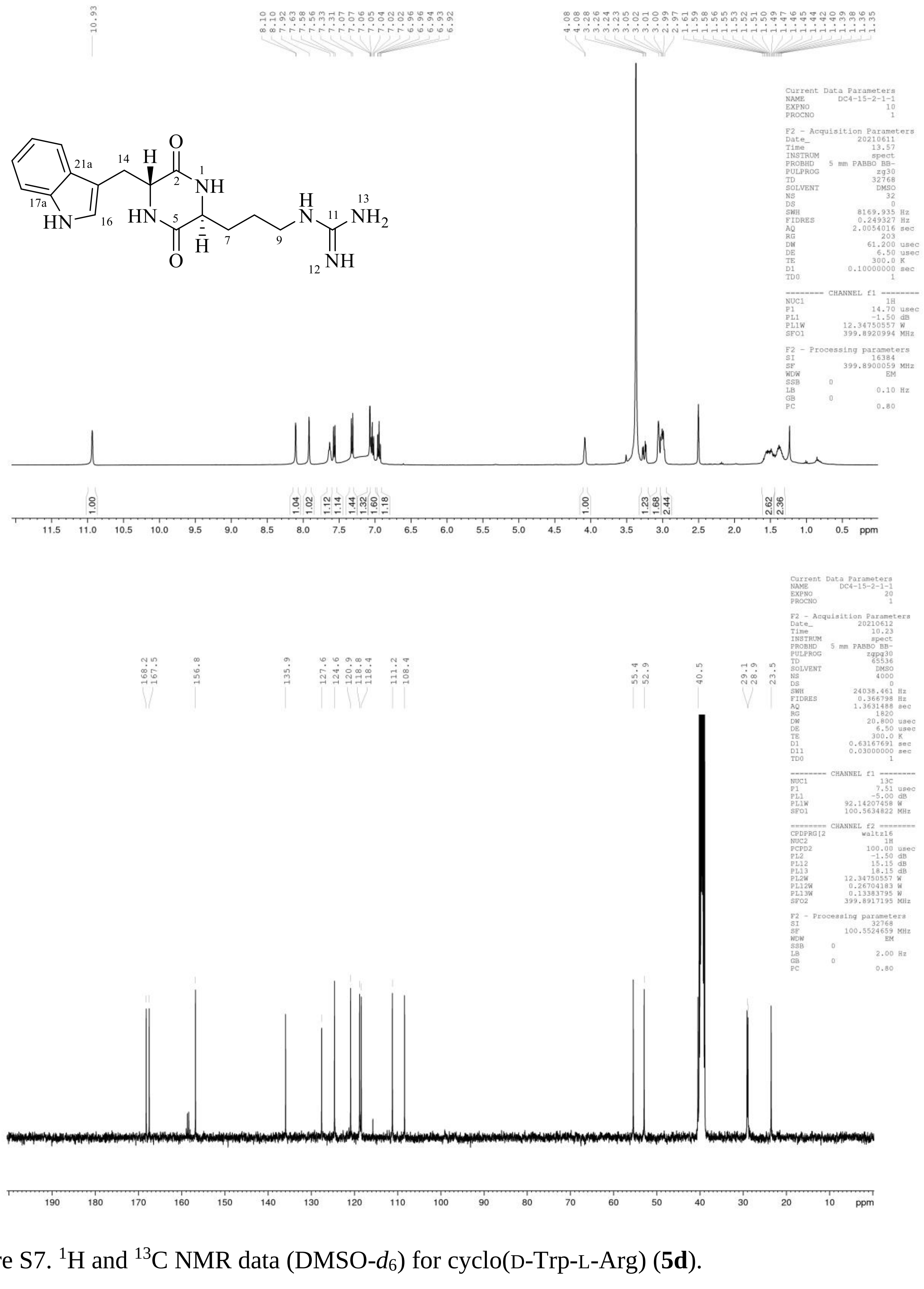

Figure S7. ^{1}H and ^{13}C NMR data (DMSO-d_6) for cyclo(D-Trp-L-Arg) (**5d**).

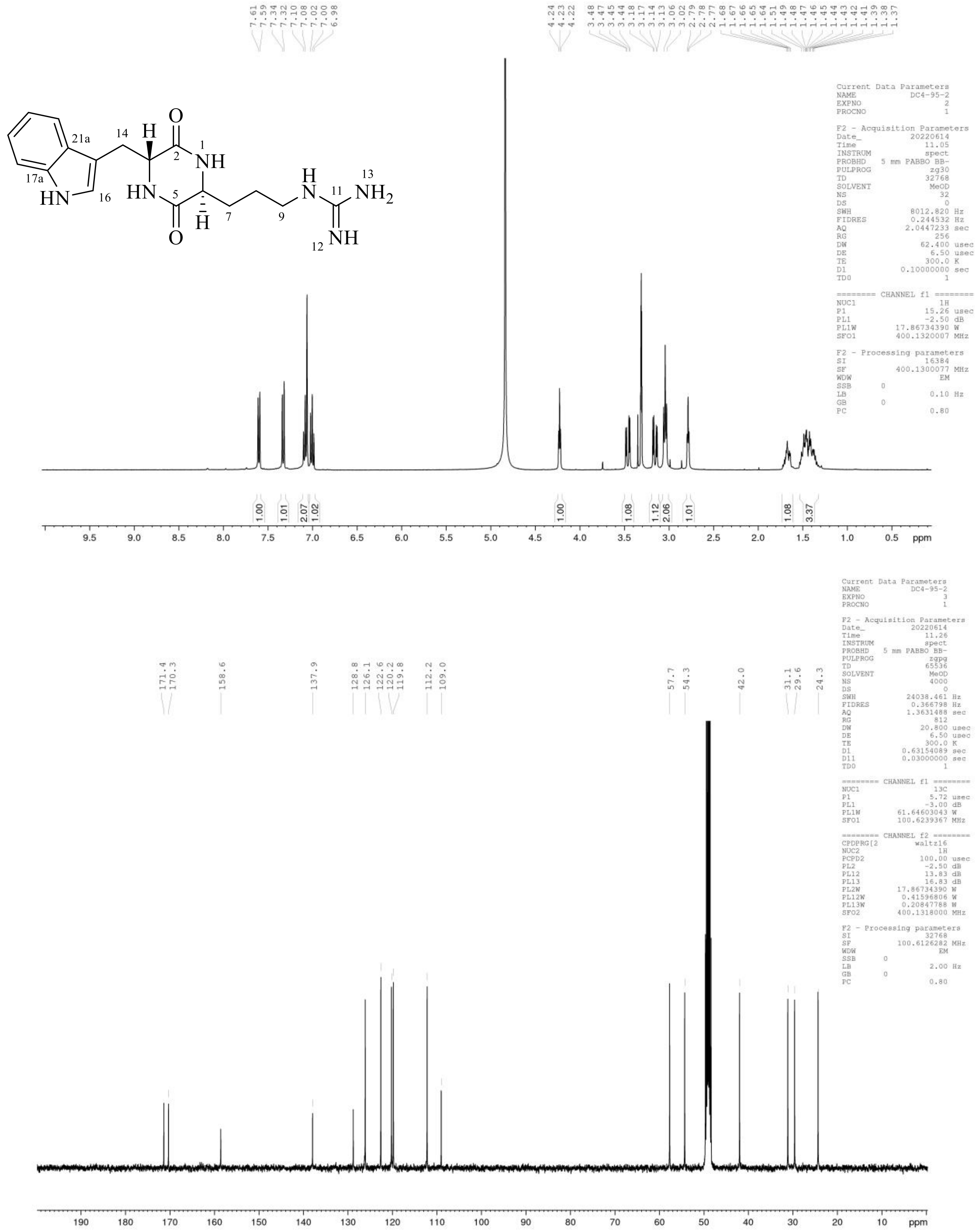

Figure S8. ^{1}H and ^{13}C NMR data (CD_3OD) for cyclo(D-Trp-L-Arg) (**5d**).

Table S1. Comparison of ^{1}H NMR chemical shifts observed[1] for **5a** and **5c** with corresponding shifts reported by Li *et al.* and calculated values of mean absolute error (MAE).

	cyclo(L-Trp-L-Arg) (**5a**)		cyclo(L-Trp-L-Arg)			cyclo(L-Trp-D-Arg) (**5c**)		cyclo(L-Trp-D-Arg)
	(current work)		Li et al.[2]			(current work)		Li et al. [2]
**Position**	**^{1}H (ppm)**[1]	**ABS(δ–δ)**	**^{1}H (ppm)**		**Position**	**^{1}H (ppm)**	**ABS(δ–δ)**	**^{1}H (ppm)**
**NH-1**	7.99	0.02	8.01		**NH-1**	7.91	0.01	7.92
**3**	4.31	0.00	4.31		**3**	4.22	0.01	4.23
**NH-4**	8.09	0.01	8.10		**NH-4**	8.10	0.01	8.11
**6**	3.69	0.01	3.68		**6**	2.77	0.00	2.77
**7a**	0.86	0.01	0.87		**7a**	1.67	0.00	1.67
**7b**	0.51	0.02	0.49		**7b**	1.43	0.01	1.44
**8a**	0.86	0.07	0.79		**8**	1.43	0.01	1.44
**8b**	0.71	0.08	0.79		**9**	3.04	0.00	3.04
**9**	2.62	0.00	2.62		**NH-10**	7.47	0.03	7.44
**NH-10**	7.23	0.02	7.21		**14a**	3.46	0.01	3.47
**14a**	3.49	0.00	3.49		**14b**	3.15	0.00	3.15
**14b**	3.14	0.00	3.14		**16**	7.06	0.00	7.06
**16**	7.08	0.00	7.08		**NH-17**	10.91	0.00	10.91
**NH-17**	10.86	0.00	10.86		**18**	7.33	0.00	7.33
**18**	7.35	0.01	7.34		**19**	7.08	0.00	7.08
**19**	7.09	0.00	7.09		**20**	7.00	0.00	7.00
**20**	7.01	0.00	7.01		**21**	7.60	0.00	7.60
**21**	7.63	0.00	7.63					
	**MAE (ppm)**[3]	0.014				**MAE (ppm)** [3]	0.005	

Footnotes.

1. ^{1}H NMR shifts acquired in CD_3OD solvent, with exchangeable NH shifts acquired in DMSO-d_6 (highlighted yellow).
2. Li et al. dx.doi.org/10.1021/jp311151h. *J. Phys. Chem. A* **2013**, *117*, 1721−1736.
3. MAE calculated using MAE = sum(ABS(δ–δ))/n, where ABS(δ–δ) is the absolute value of the difference between the chemcial shifts observed for our compound versus their compound and n is the number of values compared.

Table S2. Comparison of ^{13}C NMR chemical shifts (CD_3OD) observed for **5a** and **5c** with corresponding shifts reported by Li *et al.* and calculated values of mean absolute error (MAE).

	cyclo(L-Trp-L-Arg) (**5a**)		cyclo(L-Trp-L-Arg)		cyclo(L-Trp-D-Arg) (**5c**)		cyclo(L-Trp-D-Arg)	cyclo(L-Trp-D-Arg)
	(current work)		Li et al.[1]		(current work)		Li et al.[1]	Li et al.[1]
							**reference corrected**	**as published**
**Position**	**^{13}C ppm**	**ABS(δ–δ)**	**^{13}C ppm**		**^{13}C ppm**	**ABS(δ–δ)**	**^{13}C ppm**	**^{13}C ppm**
**2**	169.9	0.00	169.9		171.4	0.00	171.4	169.9
**3**	57.5	0.00	57.5		57.7	0.00	57.7	56.2
**5**	169.5	0.00	169.5		170.3	0.00	170.3	168.9
**6**	55.2	0.00	55.2		54.3	0.00	54.3	52.8
**7**	32.1	0.00	32.1		29.6	0.00	29.6	28.1
**8**	24.5	0.00	24.5		24.3	0.00	24.3	22.9
**9**	41.7	0.00	41.7		42.0	0.00	42.0	40.5
**11**	158.5	0.10	158.4		158.6	0.00	158.6	157.1
**14**	30.5	0.00	30.5		31.1	0.00	31.1	29.7
**15**	109.6	0.10	109.7		109.0	0.00	109.0	107.6
**16**	126.1	0.00	126.1		126.1	0.00	126.1	124.7
**17a**	137.8	0.00	137.8		137.9	0.00	137.9	136.5
**18**	112.2	0.00	112.2		112.2	0.00	112.2	110.7
**19**	122.5	0.00	122.5		122.6	0.00	122.6	121.1
**20**	120.3	0.10	120.2		120.2	0.00	120.2	118.8
**21**	120.2	0.10	120.3		119.8	0.00	119.8	118.3
**21a**	129.4	0.00	129.4		128.8	0.00	128.8	127.3
	**MAE (ppm)**[2]	0.02			**MAE (ppm)**[2]	0.00		

Footnotes.

1. Li *et al.* dx.doi.org/10.1021/jp311151h. *J. Phys. Chem. A* **2013**, *117*, 1721−1736. Examination of the original ^{13}C NMR data collected by Li et al. for cyclo(L-Trp-D-Arg) identified that the spectrum has been incorrectly referenced (offset required +1.44 ppm). Corrected data was used for the MAE calculation.
2. MAE calculated using MAE = sum(ABS(δ–δ))/n, where ABS(δ–δ) is the absolute value of the difference between the chemcial shifts observed for our compound versus their compound and n is the number of values compared.

Table S3. Comparison of ^{1}H NMR chemical shifts reported for CDP 2 (**1**) with corresponding shifts observed for **5a** (cyclo(L-Trp-L-Arg) in either DMSO-d_6 or CD_3OD (with exchangeables in DMSO-d_6) and calculated values of mean absolute error (MAE).

	CDP 2 [1]			L,L synthetic (**5a**)		L,L synthetic (**5a**)
	"DMSO-d_6"			DMSO-d_6		CD_3OD + DMSO-d_6 [2]
**Position**	**^{1}H (ppm)**	**ABS(δ–δ) (DMSO-d_6)**	**ABS(δ–δ) (CD_3OD + DMSO-d_6)**	**^{1}H (ppm)**		**^{1}H (ppm)**
**NH-1**	8.11	0.12	0.12	7.99		7.99
**3**	4.37	0.26	0.06	4.11		4.31
**NH-4**	8.17	0.08	0.08	8.09		8.09
**6**	3.74	0.18	0.05	3.56		3.69
**7a**	0.81	0.21	0.05	1.02		0.86
**7b**	0.54	0.07	0.03	0.61		0.51
**8**	0.79	0.08	0.01	0.87		0.78
**9**	2.72	0.04	0.10	2.68		2.62
**NH-10**	7.28	0.05	0.05	7.23		7.23
**14a**	3.51	0.28	0.02	3.23		3.49
**14b**	3.19	0.15	0.05	3.04		3.14
**16**	7.12	0.06	0.04	7.06		7.08
**NH-17**	10.77	0.09	0.09	10.86		10.86
**18**	7.41	0.08	0.06	7.33		7.35
**19**	7.15	0.13	0.06	7.02		7.09
**20**	7.01	0.06	0.00	6.95		7.01
**21**	7.69	0.12	0.06	7.57		7.63
	**MAE (ppm)**[3]	0.12	0.05			

Footnotes.

1. CDP 2 data from Deepa *et al.* dx.doi.org/10.3389/fmicb.2015.00876
2. 'CD_3OD+DMSO-d_6' is ^{1}H shifts observed in CD_3OD with exchangeable NH signals observed in DMSO-d_6.
3. MAE calculated using MAE = sum(ABS(δ–δ))/n, where ABS(δ–δ) is the absolute value of the difference between the chemcial shifts observed for our compound versus their compound and n is the number of values compared.

Table S4. Comparison of ^{1}H NMR chemical shifts reported for CDP 3 (**2**) with corresponding shifts observed for **5a** (cyclo(L-Trp-L-Arg) in either DMSO-d_6 or CD_3OD (with exchangeables in DMSO-d_6) and calculated values of mean absolute error (MAE).

	CDP 3 [1]			L,L synthetic (**5a**)		L,L synthetic (**5a**)
	"DMSO-d_6"			DMSO-d_6		CD_3OD + DMSO-d_6 [2]
**Position**	**^{1}H (ppm)**	**ABS(δ–δ) (DMSO-d_6)**	**ABS(δ–δ) (CD_3OD + DMSO-d_6)**	**^{1}H (ppm)**		**^{1}H (ppm)**
NH-1	8.24	0.25	0.25	7.99		7.99
3	4.43	0.32	0.12	4.11		4.31
NH-4	8.21	0.12	0.12	8.09		8.09
6	3.88	0.32	0.19	3.56		3.69
7a	0.88	0.14	0.02	1.02		0.86
7b	0.61	0.00	0.10	0.61		0.51
8	0.79	0.08	0.01	0.87		0.78
9	2.77	0.09	0.15	2.68		2.62
NH-10	7.34	0.11	0.11	7.23		7.23
14a	3.61	0.38	0.12	3.23		3.49
14b	3.33	0.29	0.19	3.04		3.14
16	7.22	0.16	0.14	7.06		7.08
NH-17	10.91	0.05	0.05	10.86		10.86
18	7.43	0.10	0.08	7.33		7.35
19	7.21	0.19	0.12	7.02		7.09
20	7.01	0.06	0.00	6.95		7.01
21	7.77	0.20	0.14	7.57		7.63
	**MAE (ppm)**[3]	0.17	0.11			

Footnotes.

1. CDP 3 data from Deepa *et al.* dx.doi.org/10.3389/fmicb.2015.00876
2. 'CD_3OD+DMSO-d_6' is ^{1}H shifts observed in CD_3OD with exchangeable NH signals observed in DMSO-d_6.
3. MAE calculated using MAE = sum(ABS(δ–δ))/n, where ABS(δ–δ) is the absolute value of the difference between the chemcial shifts observed for our compound versus their compound and n is the number of values compared.

Table S5. Comparison of ^{13}C NMR chemical shifts reported for CDP 2 (**1**) with corresponding shifts observed for **5a** (cyclo(L-Trp-L-Arg) in either DMSO-d_6 or CD_3OD and calculated values of mean absolute error (MAE).

	CDP 2 [1]			L,L synthetic		L,L synthetic
	"DMSO-d_6"			DMSO-d_6		CD_3OD
**Position**	**^{13}C**	**ABS(δ–δ) (DMSO-d_6)**	**ABS(δ–δ) (CD_3OD)**	**^{13}C**		**^{13}C**
2	169.8	2.6	0.1	167.2		169.9
3	57.2	1.7	0.3	55.5		57.5
5	169.3	2.4	0.2	166.9		169.5
6	55.2	1.8	0.0	53.4		55.2
7	32.2	1.6	0.1	30.6		32.1
8	24.6	1.2	0.1	23.4		24.5
9	41.7	1.6	0.0	40.1		41.7
11	158.5	1.8	0.0	156.7		158.5
14	30.5	1.5	0.0	29.0		30.5
15	109.6	1.0	0.0	108.6		109.6
16	126.2	1.6	0.1	124.6		126.1
17a	137.9	2.0	0.1	135.9		137.8
18	112.3	1.1	0.1	111.2		112.2
19	122.4	1.6	0.1	120.8		122.5
20	120.3	1.9	0.0	118.4		120.3
21	120.4	1.4	0.2	119.0		120.2
21a	129.4	1.6	0.0	127.8		129.4
	**MAE (ppm)**[2]	1.7	0.1			

Footnotes.

1. CDP 2 data from Deepa *et al.* dx.doi.org/10.3389/fmicb.2015.00876
2. MAE calculated using MAE = sum(ABS(δ–δ))/n, where ABS(δ–δ) is the absolute value of the difference between the chemcial shifts observed for our compound versus their compound and n is the number of values compared.

Table S6. Comparison of ^{13}C NMR chemical shifts reported for CDP 3 (**2**) with corresponding shifts observed for **5a** (cyclo(L-Trp-L-Arg) in either DMSO-d_6 or CD_3OD and calculated values of mean absolute error (MAE).

	CDP 3 [1]			L,L synthetic		L,L synthetic
	"DMSO-d_6"			DMSO-d_6		CD3OD
**Position**	**^{13}C**	**ABS(δ–δ) (DMSO-d_6)**	**ABS(δ–δ) (CD_3OD)**	**^{13}C**		**^{13}C**
2	170.1	2.9	0.2	167.2		169.9
3	59	3.5	1.5	55.5		57.5
5	170.0	3.1	0.5	166.9		169.5
6	54.7	1.3	0.5	53.4		55.2
7	32.7	2.1	0.6	30.6		32.1
8	25.5	2.1	1.0	23.4		24.5
9	42.0	1.9	0.3	40.1		41.7
11	158.8	2.1	0.3	156.7		158.5
14	31.5	2.5	1.0	29.0		30.5
15	109.7	1.1	0.1	108.6		109.6
16	127.2	2.6	1.1	124.6		126.1
17a	138.2	2.3	0.4	135.9		137.8
18	113.3	2.1	1.1	111.2		112.2
19	123.1	2.3	0.6	120.8		122.5
20	121.3	2.9	1.0	118.4		120.3
21	121.4	2.4	1.2	119.0		120.2
21a	130.3	2.5	0.9	127.8		129.4
	**MAE (ppm)**[2]	2.3	0.7			

Footnotes.

1. CDP 3 data from Deepa *et al.* dx.doi.org/10.3389/fmicb.2015.00876
2. MAE calculated using MAE = sum(ABS(δ–δ))/n, where ABS(δ–δ) is the absolute value of the difference between the chemcial shifts observed for our compound versus their compound and n is the number of values compared.
